# A hospital demand and capacity intervention approach for COVID-19

**DOI:** 10.1371/journal.pone.0283350

**Published:** 2023-05-03

**Authors:** James Van Yperen, Eduard Campillo-Funollet, Rebecca Inkpen, Anjum Memon, Anotida Madzvamuse

**Affiliations:** 1 Department of Mathematics, School of Mathematical and Physical Sciences, University of Sussex, Brighton, United Kingdom; 2 Department of Mathematics, School of Mathematical, Statistical and Actuarial Sciences, University of Kent, Canterbury, United Kingdom; 3 Department of Mathematics and Statistics, Lancaster University, Lancaster, United Kingdom; 4 Department of Primary Care and Public Health, Brighton and Sussex Medical School, Brighton, United Kingdom; 5 Department of Mathematics, University of Johannesburg, Johannesburg, South Africa; 6 Department of Mathematics, University of British Columbia, Vancouver, Canada; 7 Department of Mathematics, University of Pretoria, Pretoria, South Africa; Texas A&M University College Station, UNITED STATES

## Abstract

The mathematical interpretation of interventions for the mitigation of epidemics in the literature often involves finding the optimal time to initiate an intervention and/or the use of the number of infections to manage impact. Whilst these methods may work in theory, in order to implement effectively they may require information which is not likely to be available in the midst of an epidemic, or they may require impeccable data about infection levels in the community. In reality, testing and cases data can only be as good as the policy of implementation and the compliance of the individuals, which implies that accurately estimating the levels of infections becomes difficult or complicated from the data that is provided. In this paper, we demonstrate a different approach to the mathematical modelling of interventions, not based on optimality or cases, but based on demand and capacity of hospitals who have to deal with the epidemic on a day to day basis. In particular, we use data-driven modelling to calibrate a susceptible-exposed-infectious-recovered-died type model to infer parameters that depict the dynamics of the epidemic in several regions of the UK. We use the calibrated parameters for forecasting scenarios and understand, given a maximum capacity of hospital healthcare services, how the timing of interventions, severity of interventions, and conditions for the releasing of interventions affect the overall epidemic-picture. We provide an optimisation method to capture when, in terms of healthcare demand, an intervention should be put into place given a maximum capacity on the service. By using an equivalent agent-based approach, we demonstrate uncertainty quantification on the likelihood that capacity is not breached, by how much if it does, and the limit on demand that almost guarantees capacity is not breached.

## 1 Introduction

The resurgence of the severe acute respiratory syndrome coronavirus 2 (SARS-CoV-2) that causes COVID-19, and its mutant variants, has put national health systems in most countries under significant pressure. Throughout the pandemic, the UK government has implemented a combination of non-pharmaceutical and pharmaceutical interventions for England to combat excessive COVID-19 infections in the community [1]. The government provided resources and projections for local authorities to help manage the spread of COVID-19 within their regions and manage resources accordingly. However, at the beginning of 2022 the government announced that England would be moving into a state of “living with COVID-19”, which practically meant a removal of most rules and a reduction of services that were put in place to combat the epidemic. In particular, the removal of rules on public testing, tracking and reporting of positive COVID-19 infections, and the access to free testing kits and testing sites, dramatically changed the way data could be used for mathematical modelling and reporting. One only needs to check the weekly number of people receiving a PCR test or the number virus tests reported from February (when the change was announced) to April (when the change began) to as soon as May to see a severe drop in reporting [2].

Whilst COVID-19 tests in hospitals are still being performed routinely, the lack of community testing is problematic, especially going into winter seasons. Population health management teams in hospitals and public health intelligence teams in local authorities still need to be able to forecast the potential demand COVID-19 resurgence has on hospitals. Elective treatments, such as surgery and chemotherapy, are still substantially backlogged due to the pandemic, and another resurgence of COVID-19 could add more pressure on healthcare systems without proper planning. What this study aims to contribute are ways in which local authorities can use their own calibrated mathematical models, models which are calibrated to the data of their region, to run plausible scenarios based on effective measures to control their hospital demand and capacity. National scenario-based studies of operational importance have been conducted by modelling groups across the country to understand the impact of the UK Foot and Mouth outbreak of 2001, see [3] and references therein, and the UK SARS H1N1 pandemic of 2009, see [4] and references therein. For a mathematical modelling approach to a generic influenza pandemic at a national or international level, see [5] and references therein. Experimental scenario-based studies have been conducted using ICU capacity as a determinant [6, 7], deducing the best intervention scenario possible given some set of parameters [8, 9], and considering intervention scenarios based on the current number of infections [10–12].

The objective of this paper is twofold: one is to demonstrate the flexibility of data-driven mathematical modelling for providing robust intervention scenarios, and the other is to demonstrate the capabilities of mathematical modelling for use by local authorities. In this study we focus on hospital demand and capacity as the metric to decide if an intervention is initiated. We consider the situation whereby given the *R* number of the infectious disease, how using current hospital demand to initiate and lift an intervention can significantly change the epidemic. We describe methodology on how to use demand and capacity within a mathematical framework for healthcare planners and local authorities. This makes scenario-based forecasting accessible, whereby the scenarios could include different types of interventions based on practical and operational metrics. For operational applications, we demonstrate ways in which one can use deterministic models for quick outputs to determine optimal demand values given a certain capacity, and then use an agent-based approach to produce the uncertainty around the optimal demand. In particular, one can run scenarios to determine the likelihood that capacity will be breached when considering certain proportions of the optimal capacity, and provide this information to decision makers and resource managers.

To demonstrate the flexibility of mathematical models, we use a susceptible-exposed-infectious-recovered-died (SEIR-D) framework to produce forecasts based on different parameter values, a very standard approach in the mathematical modelling of infectious diseases literature. We do this in a deterministic way, using a system of differential equations, and in a stochastic way, using two different types of agent-based approaches. We demonstrate how one can generate a stochastic analogue of the deterministic SEIR-D models from an agent-based approach, which allows for quicker uncertainty quantification than standard agent-based approaches. We also demonstrate how one can reformulate an agent-based approach to be in terms of the length of stay. The length of stay is often an easier estimatable quantity for public health analysts to work with, since they often have patient level data. A plethora of published articles that present work using both deterministic methods and agent-based methods for forecasting COVID-19 dynamics are found here [13–22] and the references therein. The interventions described in this study are a simple change of parameters when certain conditions on the demand on hospitals are met. We estimate parameters associated to a time in lockdown in the UK and use these to represent the parameters of an intervention, and scale them to represent a time outside of an intervention. The change of parameters in this study are suggestive of a non-pharmaceutical intervention rather than pharmaceutical. This means that the interpretation of the changes of parameters could represent the change in the average number of contacts per day per person or the probably of a successful transmission, perhaps due to a new variant.

The outline of this paper is as follows. In Section 2 we introduce the SEIR-D framework, the equation-based deterministic method in Section 2.1 and the two agent-based approaches in Section 2.2. Section 3 is where we describe the intervention approaches and explore changes in parameters. Sections 3.2 to 3.4 introduce the interventions, Section 3.5 demonstrates an optimisation procedure for demand and capacity given an *R* number, and in Section 3.6 we investigate the effect stochastic perturbations have on the optimisation results. In particular, we numerically obtain the uncertainty around how often a breach happens, how much the breaches go over capacity and the differences in demand and capacity. In Section 4 we outline some limitations of this work with potential solutions and some future work, and in Section 5 we summarise the main findings of this study and conclude the paper.

## 2 Methods: SEIR-D framework

The SEIR-D framework is a well known mathematical instrument for the study of infectious diseases. Often accredited to the work of Kermack and McKendrick in 1927 [23], SEIR-D type models exploded into the forefront of mathematical models due to the pandemic. The deterministic type, which is often what individuals mean when they say SIR-type model, is a system of ordinary differential equations describing the interactions between different states an individual can be in during their infection. These states are often called compartments, and SIR-type models are thus often called compartmental models. In this setup, homogeneous populations are considered rather than individuals, which means that the compartments describe proportions of the population that smoothly move between states. They have been used in many infectious disease studies, such as for sexually transmitted diseases, respiratory diseases, gastrointestinal diseases and vector-borne diseases [24–32]. For readers unfamiliar with the subject, or readers who want to see real-life applications, we recommend the following book which has case studies on the use of SIR-type models for infectious childhood diseases, Influenza, and the West Nile virus [33]. They are often favoured in mathematical biology since they are amenable to mathematical analysis, such as long-term evolution, steady-state analysis and parameter identifiability [34–36]. Other than analysis, they are often used for scenario-based forecasts and sensitivity analysis because they are fast to run. Most programming languages have optimised libraries which solve systems of differential equations fast and extremely accurately. This speed is crucial for parameter estimation. One can often find estimates for most parameters associated to an infectious disease in other studies, such as the average incubation time, but not necessarily when it comes to parameters associated to a region, such as the average transmission rate or average admission rate. These regional parameters are crucial to the development of models that can forecast local dynamics.

On the other hand, deterministic methods lack uncertainty quantification. This is where an agent-based approach to modelling is advantageous, the stochastic part to the SEIR-D framework. Understanding how uncertain the forecast can be, by providing robust measures of scale, is critical for healthcare planners to have worst-case scenarios for resource allocation. Similar to standard compartmental models, agent-based models (also known as individual-based models) are versatile in what they can model, being used in areas such as biology, engineering, politics and economics [37–40]. The agent-based approach considers agents and their behaviours, whilst the standard deterministic approach, also referred to as equation-based modelling, focuses on observables and equations. For example, the agent-based approach can characterise variable contacts between agents in a way which is practically impossible to describe at a population level. Due to the nature of infectious diseases, having recognisable transmissions between distinct states, agent-based approaches have slowly been making a surge to becoming the standard method for mathematical epidemiological modelling [41–45]. However, agent-based approaches often lack speed. This is due to evaluating all agent’s behaviours at each time step, which is computationally expensive when considering large population sizes. In this study, we reformulate the agent-based approach so that it can be represented as a branching model by collect similar agents together into a single state. We then only need to execute the expressions for these states, which is significantly quicker. Nevertheless, agent-based approaches were widely used during the COVID-19 pandemic for policy decisions due to their flexibility. For example, the famed Imperial College Model [16], also known as CovidSim, is an agent-based model based on previous works conducted on modelling influenza pandemics [5, 46, 47]. Other widely used agent-based models of COVID-19 are covasim and OpenABM-Covid19 [48–51].

In the SEIR-D framework, the underlying assumption is that all compartments follow an exponential distribution, that is to say that an agent’s length of stay in each compartment is an exponentially distributed length of time. This is the result of the underlying dynamics of an infectious disease being modelled as a continuous-time Markov chain [52–54]. In the deterministic setup, this is seen because the rates of movement between compartments are proportional to the size of the compartment, which leads to a solution involving the exponential function in some capacity. Given the wide ranging impact and applications of SEIR-D models, it seems reasonable that infectious diseases follow the rules of a continuous-time Markov chain, however it is not necessarily clear if this assumption is valid for patients in hospital. If the length of stay in hospital is not exponentially distributed, then it seems that the SEIR-D framework is not suitable. In this study we have reformulated an agent-based model that uses the length of stay distribution directly in the model, and verified it works by comparing it to the outputs of the SEIR-D model. That means if the length of stay is not exponentially distributed, or not even a related distribution like the Erlang distribution, then there exists a setup that can utilise this information.

**Fig 1:**
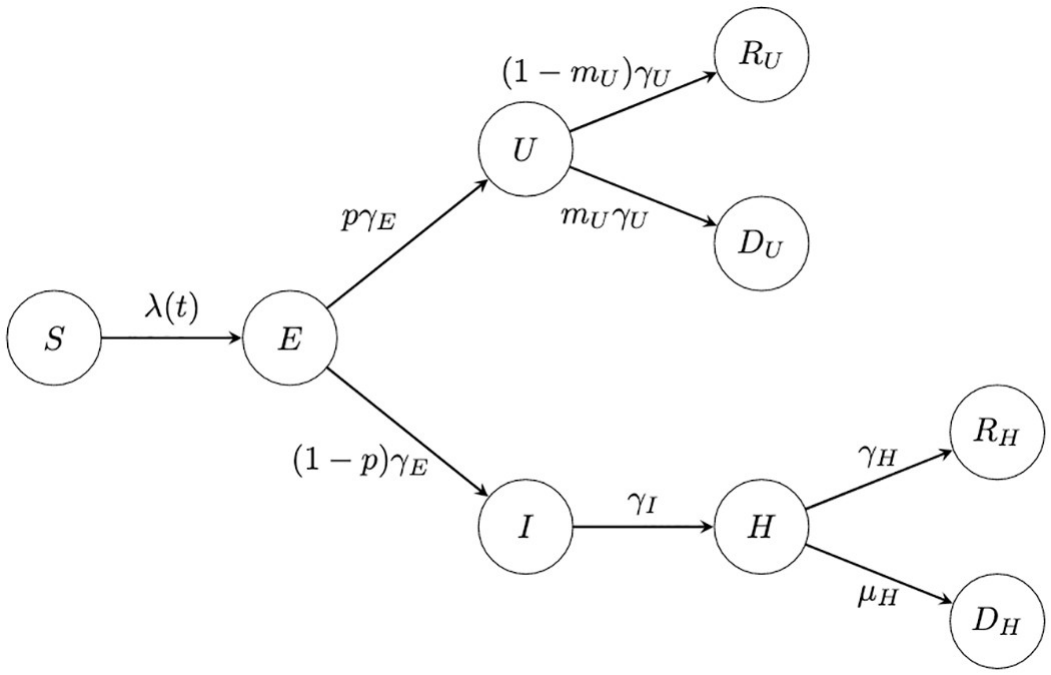
Schematic representation of the compartmental pathways on which results in the equation-based SEIR-D mathematical model.

**Fig 2:**
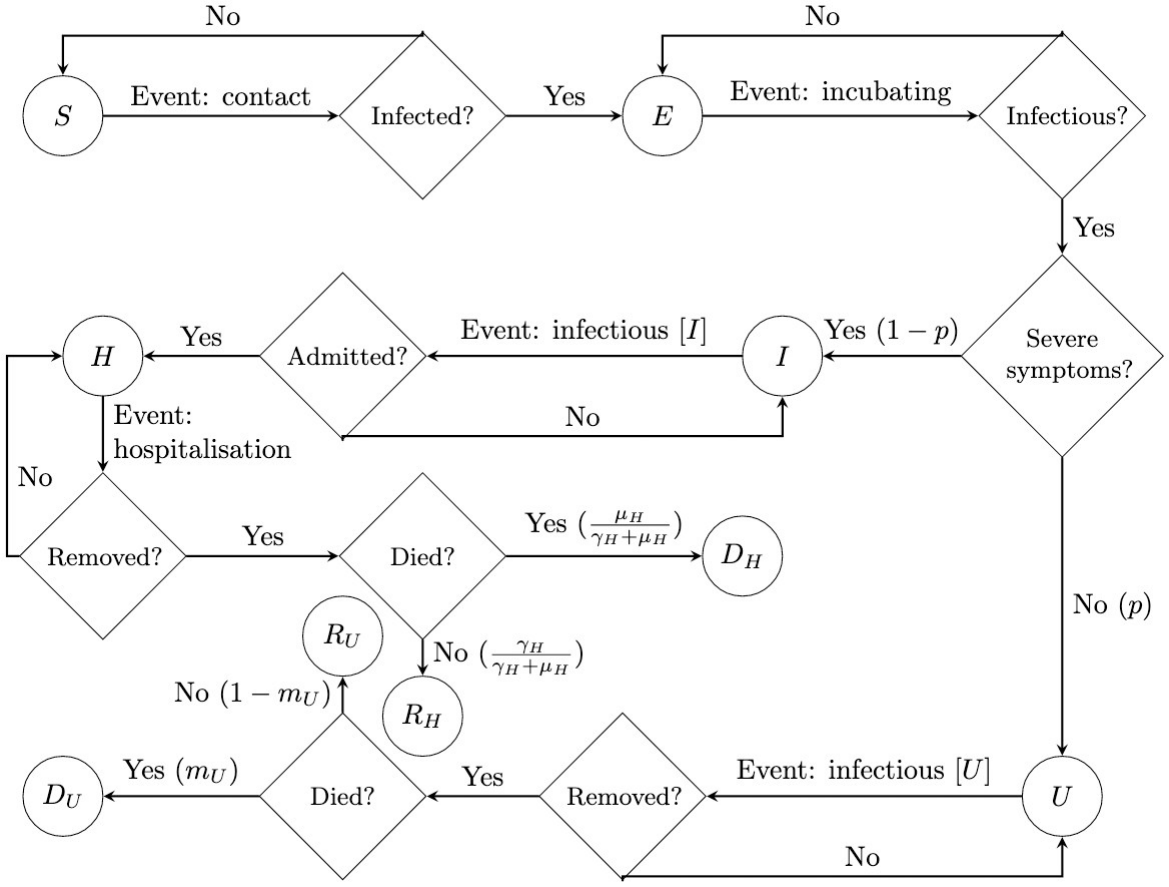
Schematic representation of the flowchart which results in the agent-based mathematical model.

The schematics for the deterministic model and the agent-based model are presented in Figs 1 and 2 respectively. The schematic in Fig 1 is a very standard representation of an SEI*R-D model, whereby the infectious compartment has been split in two to represent the different characteristics of the individuals in the data. The schematic in Fig 2 is a flowchart, which can be used as a representation of any workflow or process. In both, each of the compartments are labelled by the different states of an individual who is susceptible, infected or recovered with the infectious disease at hand. The continuous flow of the deterministic model is depicted by the lack of decision stations, whereby the letter on top of the arrows represent the rate of moving between compartments. The decision states in the agent-based approach represent the Bernoulli trial each agent has to make at each time point, with a probability associated if a fork of states exists after a decision.

For illustrative purposes, we only consider the geographical regions of North West England, South East England and the nation of England. We chose these regions as both National Health Service (NHS) region and local authority region are the same, but in principle this work can be conducted for any area with appropriate datasets.

### 2.1 Equation-based modelling

The equation-based model is a dynamic system of ordinary differential equations, governed by the interactions depicted in Fig 1, supported by non-negative initial conditions

$$\dot{S}=-\beta \frac{U+I}{N}S,\;\;\;\;\;\;t\in \left(0,T\right],\;S\left(0\right)=S_{0},$$ (1)

$$\dot{E}=\beta \frac{U+I}{N}S-\gamma_{E}E,\;\;t\in \left(0,T\right],\;E\left(0\right)=E_{0},$$ (2)

$$\dot{U}=p\;\gamma_{E}E-\gamma_{U}U,\;\;\;\;\;\;t\in \left(0,T\right],\;U\left(0\right)=U_{0},$$ (3)

$$\dot{I}=\left(1-p\right)\gamma_{E}E-\gamma_{I}I,\;\;\;\;\;t\in \left(0,T\right],\;I\left(0\right)=I_{0},$$ (4)

$$\dot{H}=\gamma_{I}I-\left(\gamma_{H}+\mu_{H}\right)H,\;\;t\in \left(0,T\right],\;H\left(0\right)=H_{0}$$ (5)

$${\dot{R}}_{U}=\left(1-m_{U}\right)\gamma_{U}U,\;\;\;\;\;t\in \left(0,T\right],\;R_{U}\left(0\right)=R_{U,0},$$ (6)

$${\dot{R}}_{H}=\gamma_{H}H,\;\;\;\;\;\;t\in \left(0,T\right],\;R_{H}\left(0\right)=R_{H,0},$$ (7)

$${\dot{D}}_{U}=m_{U}\gamma_{U}U,\;\;\;t\in \left(0,T\right],\;D_{U}\left(0\right)=D_{U,0},$$ (8)

$${\dot{D}}_{H}=\mu_{H}H,\;\;\;\;\;t\in \left(0,T\right],\;D_{H}\left(0\right)=D_{H,0}.$$ (9)

For ease of reference, we shall refer to this system of equations as the SEIR-D model. Here, the dot above the notation denotes the time derivative. In this setting, *N* denotes the total regional population. *S*(*t*) denotes the proportion of the total population *N* who are susceptible to the disease, COVID-19. Susceptible individuals become exposed to the disease, i.e. they are carrying the disease but are not currently infectious, to form the *E*(*t*) subpopulation. A 100(1 − *p*)% proportion of *E*(*t*) become infectious and will require hospitalisation in the future, denoted *I*(*t*). They soon become the hospitalised, denoted by *H*(*t*). A 100*m*_*H*_% proportion of *H*(*t*) die, denoted *D*_*H*_(*t*), where

$$\begin{array}{c} m_{H}≔\frac{\mu_{H}}{\gamma_{H}+\mu_{H}}, \end{array}$$

and the remaining proportion are discharged, denoted *R*_*H*_(*t*). The remaining proportion of *E*(*t*) become infectious and will not require hospitalisation, denoted *U*(*t*). A 100*m*_*U*_% proportion of *U*(*t*) die, denoted *D*_*U*_(*t*), and the remaining proportion recover, denoted *R*_*U*_(*t*).

As is standard for epidemiological models of this nature, λ(*t*) denotes the average infectivity rate and takes the form

$$\begin{array}{c} \lambda \left(t\right)≔\beta \;\frac{U(t)+I(t)}{N}, \end{array}$$

where *β* denotes the average transmission rate, and the remainder describes the average probability of meeting an infectious person. $\gamma_{E}^{-1}$ denotes the average incubation time, $\gamma_{U}^{-1}$ denotes the average infectious period for those not needing hospital treatment, $\gamma_{I}^{-1}$ denotes the average infectious period from becoming infectious to being admitted to hospital, $\gamma_{H}^{-1}$ denotes the average hospitalisation period for those who recover and $\mu_{H}^{-1}$ represents the average hospitalisation period for those who die. We note that *γ*_*H*_ and *μ*_*H*_ are closely related to the length of stay in hospitals. For this model, using the method of next generation matrices [55], we derive the formula for the basic reproduction number $R_{0}$ as

$$\begin{array}{c} R_{0}≔\beta (\frac{p}{\gamma_{U}}+\frac{1-p}{\gamma_{I}}). \end{array}$$ (10)

Using this, we can calculate the effective reproduction number $R_{t}$ since

$$\begin{array}{c} R_{t}≔R_{0}\;\frac{S(t)}{N}. \end{array}$$ (11)

The effective reproduction number $R_{t}$ is often referred to as the *R* number. Whilst mathematically the *R* number does not provide much information other than a classification and a comparative statistic, it was nevertheless an important quantity that governments, and the local authorities, used at the beginning of the pandemic in reporting and intervention planning. By tying the *R* number to the impact on hospitalisations, we demonstrate here how planning the outcome of an intervention changes depending on hospital resources (in a loose manner).

The data obtained and calibration method used to estimate the parameters outlined above are described in S1 Appendix. We consider the data starting from the first day of lockdown, and so we also need to infer the initial conditions. We have not yet conducted a formal investigation into the resulting log-likelihood, but it is clear that there is a continuous dependence between initial conditions and the parameters, see [56] for a comprehensive discussion. In practice, we see this manifest as an issue to calibrate *p*: if the first guess of initial conditions and parameters is not close to the “true” values, then it is *p* which changes in value the most. Mathematically, this means that *p* is sensitive to change. Although in reality *p* is characterised by how COVID-19 affects individuals from different demographics, such as age, ethnicity, and gender, we speculate this value should not change drastically between regions. In view of this, in the calibration process we fix *p* to be the same for the three regions. The standard approach for the calibration of equation-based models like this is to assume when the initial infection occurred. This is usually much earlier than the first datapoint is collected, so information on the *R* number is used to calibrate the parameters up to the point of lockdown. See studies such as [16, 57], and the references therein.

**Fig 3:**
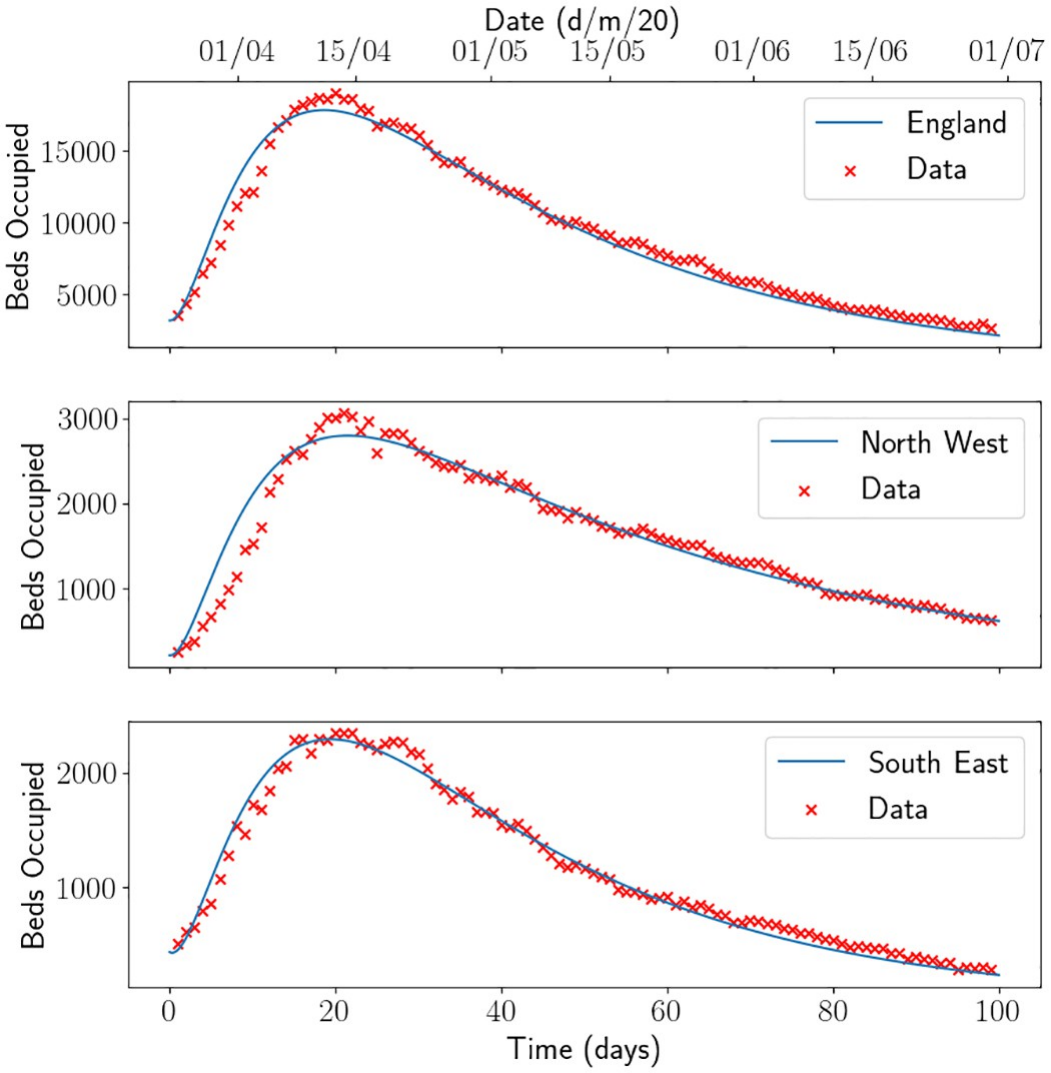
Beds occupied fit for England, South East and North West. The lines depict the output of the SEIR-D model.

**Fig 4:**
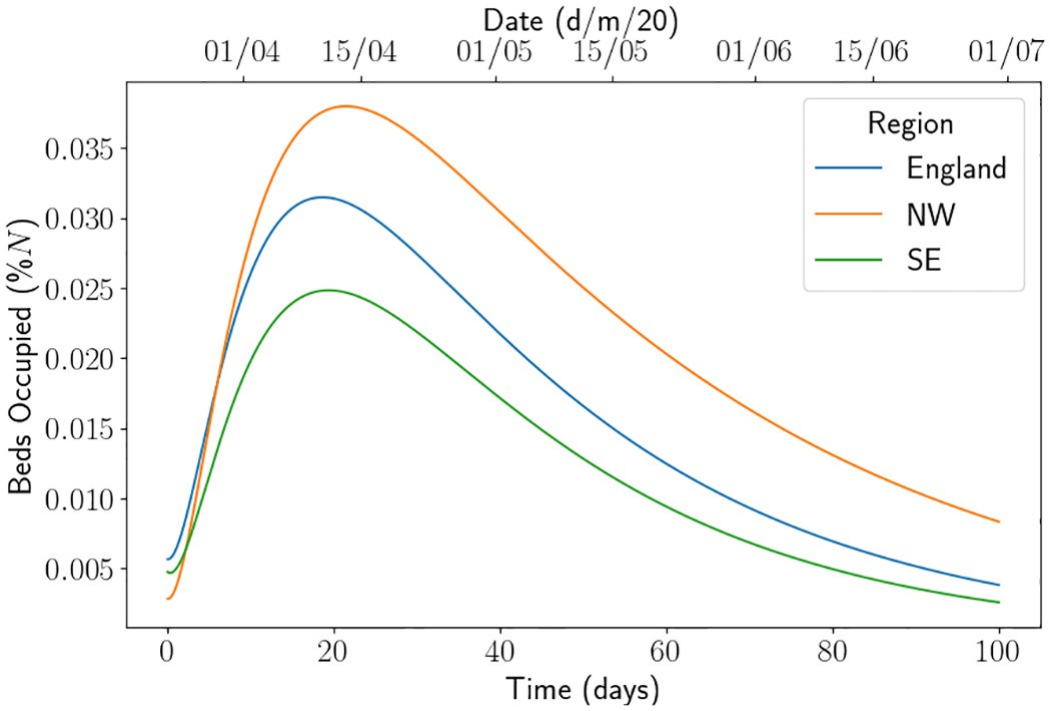
Beds occupied as a proportion of the population size for England, North West and South East. Here North West is abbreviated to NW and South East is abbreviated to SE.

**Fig 5:**
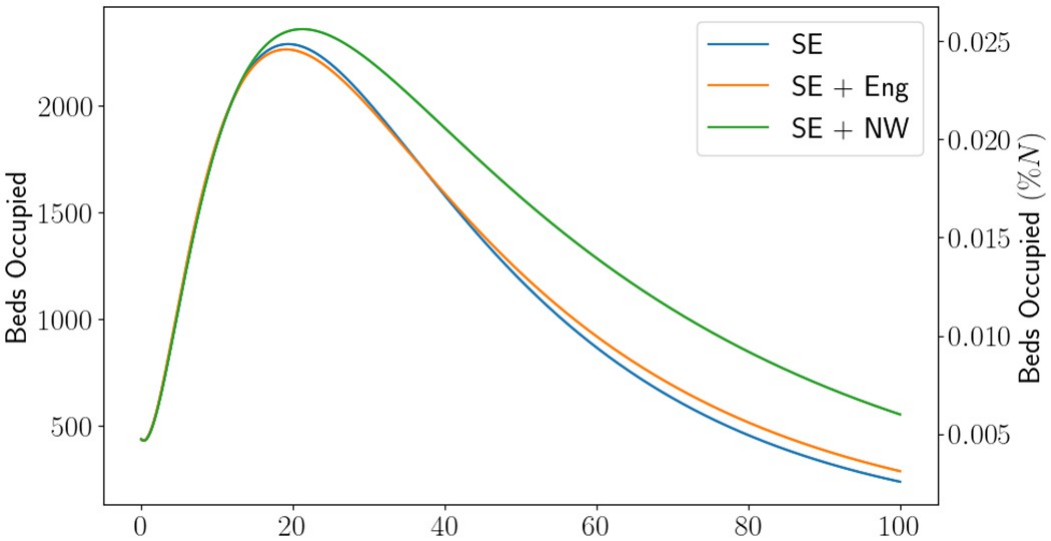
Beds occupied using the South East initial conditions but each line depicts the use of different parameters: SE is the South East parameters, SE+Eng is the the England parameters and SE+NW is the North West parameters. Here North West is abbreviated to NW and South East is abbreviated to SE.

Using the calibration procedure described in S1 Appendix, we obtain the parameters in Table 1 and initial conditions in Table 2, with a demonstration of the fit for beds occupied in each region demonstrated in Fig 3. The equivalent figure for the fit of the Sussex region, a region within the South East, can be found in Fig 2 in [13]. We note that the infected fatality ratio and average hospitalisation period for those who recover are similar across all regions, but the average hospitalisation period for those who die and the value of $R_{t}$ when the lockdown commenced are quite different. The varied values of $R_{t}$ could be explained by the amount of infections in each region. It was reported at the time that Sussex and the South East escaped quite lightly on the number of infections, whilst the North of England did not. This can be seen in Fig 4, where we have standardised the beds occupied in each region by their population size. Proportionally, the North West saw almost double the amount of patients as the South East. The higher value of $R_{t}$ for North West can also be seen by looking at the gradient of the beds occupied. There could be a myriad of reasons for this, such as the demographic of the population or the geography of the region. One should also note that, as England came out of the first lockdown and went into the first iteration of the tiered system, it was cities in the north which first started to show signs of resurgence (such as Manchester). Using *γ*_*H*_ and *μ*_*H*_ for each region, one can calculate that the estimate of the probability of discharge for England, South East and North West was 58.03%, 61.57%, 55.31% respectively. One may speculate that this is due to the number of patients in the hospitals being higher in the North West which puts pressure on the health system. We emphasise the need for parameter estimation and modelling using local data in Fig 5, where we have used the initial conditions estimated for the South East region and the parameters for the other regions. Although the parameter set for England produces a forecast close to the South East forecast (a maximum difference of approximately 60 beds), the North West parameters produce quite a dramatically different forecast (with a difference of 420 beds). It is not hard to reason why the use of local data and calibration techniques are of operational importance.

**Table 1:** Parameters of interest derived using method in [13] for three different regions of the UK.

Parameter	England	North West	South East
$R_{t}$	0.763	0.835	0.733
$\gamma_{H}^{-1}$	13.11 days	13.28 days	13.20 days
*m*_*U*_	0.0013	0.0010	0.0013
$\mu_{H}^{-1}$	18.13 days	16.43 days	21.16 days

**Table 2:** Initial conditions of interest derived using method in [13] for three different regions of the UK.

Initial Condition (%*N*)	England	North West	South East
*E*_0_	0.8912%	1.0614%	0.7096%
*U*_0_	0.0245%	0.0254%	0.0177%
*I*_0_	0.0041%	0.0016%	0.0011%

### 2.2 Agent-based modelling

Let ${\left(A\right)}_{i=1}^{N}$ denote the set of agents in the system, whereby each agent is in a state denoted *S*, *E*, *U*, *I*, *R*_*U*_, *R*_*H*_, *D*_*U*_ or *D*_*H*_. Then, for each time unit of the simulation, each agent runs a Monte Carlo simulation to determine whether they can change state according to some probability. For ease of exposition, we consider the time unit of the simulation to be days. Then, each day we can collect information about the number of individuals in each state by counting the number of agents.

In this study, we demonstrate two ways to run the Monte Carlo simulation, one using the standard Bernoulli trial approach and one using the length of stay approach. The differences arises on when a Monte Carlo trial is conducted, in the former it is conducted at each time step, whilst for the later some are completed at each time point and some are completed upon a change in state. The former is the standard method of conducting agent-based approaches, whilst the later, to the best of our knowledge, is a variation we are introducing here.

#### 2.2.1 Branching model

In the standard agent-based modelling approach, a Bernoulli trial is run at each time point to determine whether an agent changes state. In practice, this means at each time point a trial is run for each agent, where the success probability for the trial is determined by the state of the agent and event being trialled. For a particular event, the success probability is often assumed to be the same for all agents, such as the probability of turning infectious whilst incubating, but it can also be agent-dependent. For example, the probability could be characteristic-dependent, such as the probability of recovery being different for different ages, or it could be behaviour-dependent, such as being dependent on the number of contacts the agent makes per day. In the instance of characteristic-dependence, the possible states the agents could take would include this certain characteristic, such as *S*_0_ and *S*_1_ representing susceptible individuals in age-group 0 and age-group 1 respectively.

In this study we assume that all the agents have the same characteristics. As is the nature of infectious disease models, the events depicted in Fig 2 can be split into two categories, transmission and progression. Since we are not assuming different agent-characteristics, it is easy to reason that progression events are agent-independent. However, the transmission event (event: contact) is not agent independent since it depends on the number of contacts each agent makes. Denote *κ*_*i*_ to be the number of contacts susceptible agent *i* makes with an infectious agent, namely an agent in state *U* or *I*. Then, the success probability *d*_*i*_ of the Bernoulli trial to see if agent *i* becomes infected is

$$\begin{array}{c} d_{i}=1-{\left(1-a\right)}^{\kappa_{i}}, \end{array}$$ (12)

where *a* denotes the probability of a successful transmission on any contact. This is derived from a geometric distribution *X* ∼ Geo(*a*), where *X* denotes a successful transmission, which gives $d_{i}=P\left(X\leq \kappa_{i}\right)$ since we need at most 1 of the *κ*_*i*_ contacts to be a successful transmission. To form the branching model, we need all success probabilities to be agent-independent so we can collect the agents into their relevant states. We approximate (12) by

$$\begin{array}{c} d=1-(1-a\frac{U(t)+I(t)}{N}{)}^{C}, \end{array}$$ (13)

where *C* depicts the average number of contacts per day and (*U*(*t*) + *I*(*t*))*N*^−1^ approximates the proportion of *C* which are with an infectious individual. Let S(t) denote the set of indices of (*A*)_*i*_ whereby the agent is susceptible, and let S(t)=|S(t)|, i.e. the number of agents who are susceptible. Now, we can see that

$$\begin{array}{c} S\left(t+1\right)=\sum_{i\in S(t+1)}1=\sum_{i\in S(t)}1-\text{Ber}\left(d\right)=S\left(t\right)-\text{Bin}\left(S\left(t\right),d\right), \end{array}$$

where we have used the fact that the sum of Bernoulli random variables is a Binomial random variable. The second equals follows from that an agent is infected if the Bernoulli trial comes back successful (i.e. 1), so we want to count the number of failures. This result makes intuitive sense, since it states that the number of susceptible agents at time *t* + 1 is the number of previous susceptible agents at time *t* minus those who have been infected. Since all the other events are agent-independent, we can set up the same style of equation for each of the compartments, whereby *d* is set to the appropriate rate parameter associated to the event, e.g. *γ*_*E*_ for event: incubating.

Before we progress, we need to address the time step. The interpretation of the parameters we give here is that, for example, an agent has a probability *γ*_*E*_ each day of becoming infectious. However this would mean that we are approximating a continuous-time model with a coarse discrete-time model. That is to say that our model expects infections to happen only every midnight. Ideally, we need to run many Bernoulli trials per day to approximate the continuous process effectively. Let Δ*t* denote a fraction of a day, e.g. Δ*t* = 0.5 would be half one day, then we let all parameters associated to events be multiplied this value. For example, an agent would have a probability of *γ*_*E*_ Δ*t* each Δ*t* day of becoming infectious. For event: contact, this instead takes the form

$$\begin{array}{c} d_{\Delta t}≔1-(1-a\frac{U(t)+I(t)}{N}{)}^{C\Delta t}. \end{array}$$

Hence, for *t* ∈ {0, Δ*t*, …, *T* − Δ*t*}, we see that we can express the events depicted in Fig 2 by

$$S(t+\Delta t)=S\left(t\right)-s\left(t\right),\;\;\;\;\;S\left(0\right)=S_{0},$$ (14)

$$E(t+\Delta t)=E\left(t\right)+s\left(t\right)-e\left(t\right),\;\;\;\;E\left(0\right)=E_{0},$$ (15)

$$U(t+\Delta t)=U\left(t\right)+e_{U}\left(t\right)-u\left(t\right),\;\;\;\;\;U\left(0\right)=U_{0},$$ (16)

$$I(t+\Delta t)=I\left(t\right)+[e\left(t\right)-e_{U}\left(t\right)]-i\left(t\right),\;I\left(0\right)=I_{0},$$ (17)

$$H(t+\Delta t)=H\left(t\right)+i\left(t\right)-h\left(t\right),\;\;\;\;H\left(0\right)=H_{0}$$ (18)

$$R_{U}\left(t+\Delta t\right)=R_{U}\left(t\right)+[u\left(t\right)-u_{D}\left(t\right)],\;\;\;\;R_{U}\left(0\right)=R_{U,0},$$ (19)

$$R_{H}\left(t+\Delta t\right)=R_{H}\left(t\right)+[h\left(t\right)-h_{D}\left(t\right)],\;\;\;R_{H}\left(0\right)=R_{H,0},$$ (20)

$$D_{U}\left(t+\Delta t\right)=D_{U}\left(t\right)+u_{D}\left(t\right),\;\;\;\;D_{U}\left(0\right)=D_{U,0},$$ (21)

$$D_{H}\left(t+\Delta t\right)=D_{H}\left(t\right)+h_{D}\left(t\right),\;\;\;D_{H}\left(0\right)=D_{H,0},$$ (22)

where

$$\begin{array}{cc} s(t) & =\text{Bin}(S\left(t\right),d_{\Delta t}),\\ e(t) & =\text{Bin}(E\left(t\right),\gamma_{E}\Delta t),\\ e_{U}\left(t\right) & =\text{Bin}(e(t),p),\\ u(t) & =\text{Bin}(U\left(t\right),\gamma_{U}\Delta t),\\ i(t) & =\text{Bin}(I\left(t\right),\gamma_{I}\Delta t),\\ h(t) & =\text{Bin}(H\left(t\right),\gamma_{H}\Delta t+\mu_{H}\Delta t),\\ u_{D}\left(t\right) & =\text{Bin}(u\left(t\right),m_{U}),\\ h_{D}\left(t\right) & =\text{Bin}(h\left(t\right),m_{H}). \end{array}$$

We have presented the equations in this manner to emphasise the fact that only the above binomial expressions need to be computed at each time step (once) and then used in equations Eqs (14) to (22). This maintains the assumption that the population size is constant over time, as in the SEIR-D model. We refer Eqs 14 to 22 as a branching process because it is an example of a discrete-time Galton-Watson process, famously used to study the extinction of family names [58]. These have far reaching applications in the mathematical modelling of cellular aging, such as in PCR testing and human DNA evolution, and within epidemiology, such as vertical transmission models [59–65].

As one may expect, there are striking similarities between the branching process and the SEIR-D model. One can in fact derive Eqs (1) to (9) by taking the expectation of Eqs (14) to (22) and applying a limiting process as Δ*t* → 0. This leaves the equations with pairwise expectations, so the mean-field approximation (assuming *N* is large enough) is often employed to show convergence, which results in E[S(t)U(t)]≈E[S(t)]E[U(t)] and E[S(t)I(t)]≈E[S(t)]E[I(t)] [66, 67]. This approximation is often used to show that the variance of agent-based approaches tends to zero as *N* increases [68, 69]. The mean-field assumption is sometimes referred to as the thermodynamic limit. This approximation typically breaks down when the initial conditions *U*_0_ and *I*_0_ are sufficiently small so that the probability of the infectious disease dying out before becoming an epidemic is large. This is another reason why we do not use the first case reported as the initial seed time for an infection, because the resulting mean from the agent-based approach will not resemble the output from the SEIR-D model. For parameters chosen so that $R_{0}>1$, the SEIR-D model guarantees an epidemic, whilst this is not the case for the agent-based approaches. In this scenario, it is more reasonable to use an agent-based approach close to the beginning of an epidemic to incorporate the uncertainty of an outbreak. The SEIR-D model will become more useful once the epidemic is happening. In the limiting process, we also see that *β* = *Ca*.

**Fig 6:**
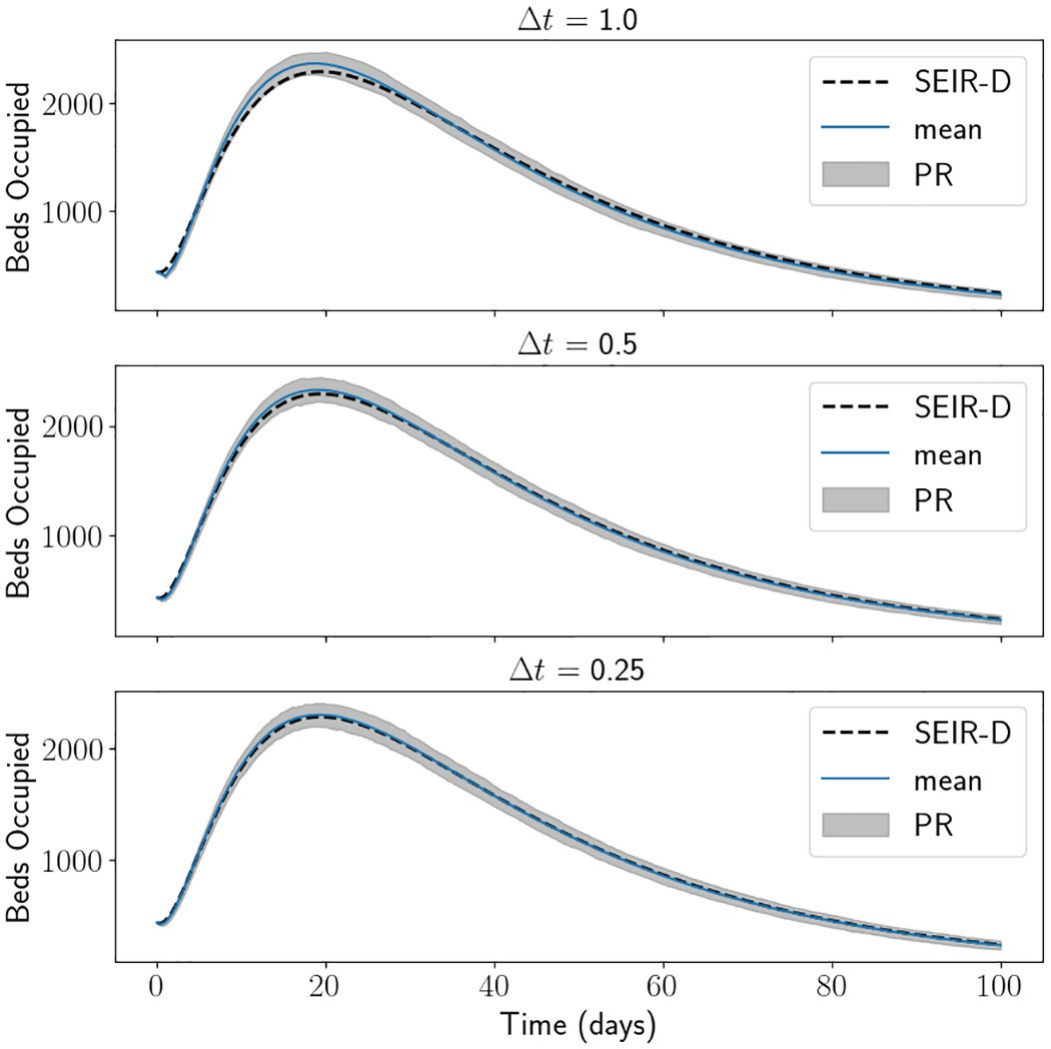
Beds occupied using the branching process, using South East parameters and initial conditions with varied values for Δ*t*. The mean of the branching process matches the SEIR-D results well for all values of Δ*t*. PR stands for percentile range.

Fig 6 is a comparison of the SEIR-D model with the branching model when using the South East parameters and initial conditions defined in Tables 1 and 2 respectively, taking several different values for Δ*t*. Here we have used 1000 Monte Carlo simulations of the branching process, which took between 1 to 3 seconds to run. We assume that *C* = 9 and then calculate *a* ≔ *βC*^−1^. As expected, as Δ*t* decreases the mean of the branching process starts to converge towards the output from the SEIR-D model. We see that the branching process overestimates the number of beds occupied compared to the SEIR-D model. We speculate this is because the rates are numerically being rounded down to the closest multiple of Δ*t*, which would mean the length of stay for each agent in each state is longer. This effect reduces as Δ*t* reduced. Note that the mean-field approximation does not break down here because the initial conditions are sufficiently large. We also demonstrate the range corresponding to 1-percentile and the 99-percentile of the simulations, named PR in the figure (short for percentile range). The PR does not seem that large and seems independent of Δ*t*, indeed the interpercentile range is around 240 (compared to 2300 beds occupied) at its maximum.

#### 2.2.2 Length of Stay model

In order to derive the length of stay model from the standard agent-based approach, we consider the same setup as the branching process up to the derivation of Eq (12). In the model, we use Eq (12) rather than Eq (13), but we do not treat the progression events as Bernoulli trials. Instead, upon an agent changing state, we stochastically generate the associated length of stay for the agent in the new state. Then, when the time duration has passed, the agent changes state according to Fig 2. Since we are comparing the outputs to the SEIR-D model, we set

$${\text{LoS}}_{E}∼\text{Exp}(\gamma_{E}),$$ (23)

$${\text{LoS}}_{U}∼\text{Exp}(\gamma_{U}),$$ (24)

$${\text{LoS}}_{I}∼\text{Exp}(\gamma_{I}),$$ (25)

$${\text{LoS}}_{H}∼\text{Exp}(\gamma_{H}+\mu_{H}),$$ (26)

where *LoS* stands for Length of Stay. Using this approach, if length of stay was not distributed exponentially, then only Eqs (23) to (26) need updating. Indeed, it was suggested that the incubation time for COVID-19 is best described by a gamma distribution [70, 71]. In the SEIR-D model we would need to approximate the gamma distribution by an Erlang distribution (make the shape parameter an integer) and then add that number of extra equations [72]. Using the length of stay approach, if there is a decision after an event, such as the infectious severity, then we need to run a Bernoulli trial with the associated probability. As for event: transmission, in order to generate *κ*_*i*_ we assume that *κ*_*i*_ ∼ Poi(*C*Δ*t*), where *C* is the average number of contacts an individual makes per day.

Again, we note that we are in fact approximating a continuous-time probability model using a discrete-time model. The discrete-time approximation did not have a huge impact on the branching model, but it does have a more severe impact on the output of the length of stay model, as seen in S1 Appendix. This is because, in general, the length of stay generated for each agent will not be a multiple of Δ*t*, yet this is what we enforce. We observe computationally that the estimated length of stay parameters from Monte Carlo simulations are in fact wrong by approximately 0.5Δ*t*. Whilst we start to see close agreement between the two sets of results as we decrease Δ*t*, the simulations take significantly longer. Indeed, Δ*t* = 1 takes approximately 7 seconds for one simulation whilst Δ*t* = 0.1 takes approximately 1 minute. Hence, it is imperative that we obtain accurate results using a larger Δ*t*. We adjust the rate parameters by adding a correction term and sampling the length of stay from this adjusted distribution, namely

$$\begin{array}{cc} {\text{LoS}}_{E} & ∼\text{Exp}(\gamma_{E}+c\left(\gamma_{E};\Delta t\right)),\\ {\text{LoS}}_{U} & ∼\text{Exp}\left(\gamma_{U}\right)+c\left(\gamma_{U};\Delta t\right)),\\ {\text{LoS}}_{I} & ∼\text{Exp}\left(\gamma_{I}\right)+c\left(\gamma_{I};\Delta t\right)),\\ {\text{LoS}}_{H} & ∼\text{Exp}\left(\gamma_{H}+\mu_{H}\right)+c\left(\gamma_{H}+\mu_{H};\Delta t\right)), \end{array}$$

where the correction term takes the form

$$\begin{array}{c} c\left(\gamma ;\Delta t\right)=\frac{\gamma^{2}\Delta t}{2-\gamma \Delta t}. \end{array}$$ (27)

For numerical justification of this correction term, see S1 Appendix. We note that Δ*t* needs to be chosen appropriately so that the denominator in *c* is non-zero. However, this is a reasonable assumption since typically *γ* < 1 (as it is a rate and its reciprocal in normally larger than one) and Δ*t* ≤ 1, since we typically consider simulating each day or several simulations per day.

**Fig 7:**
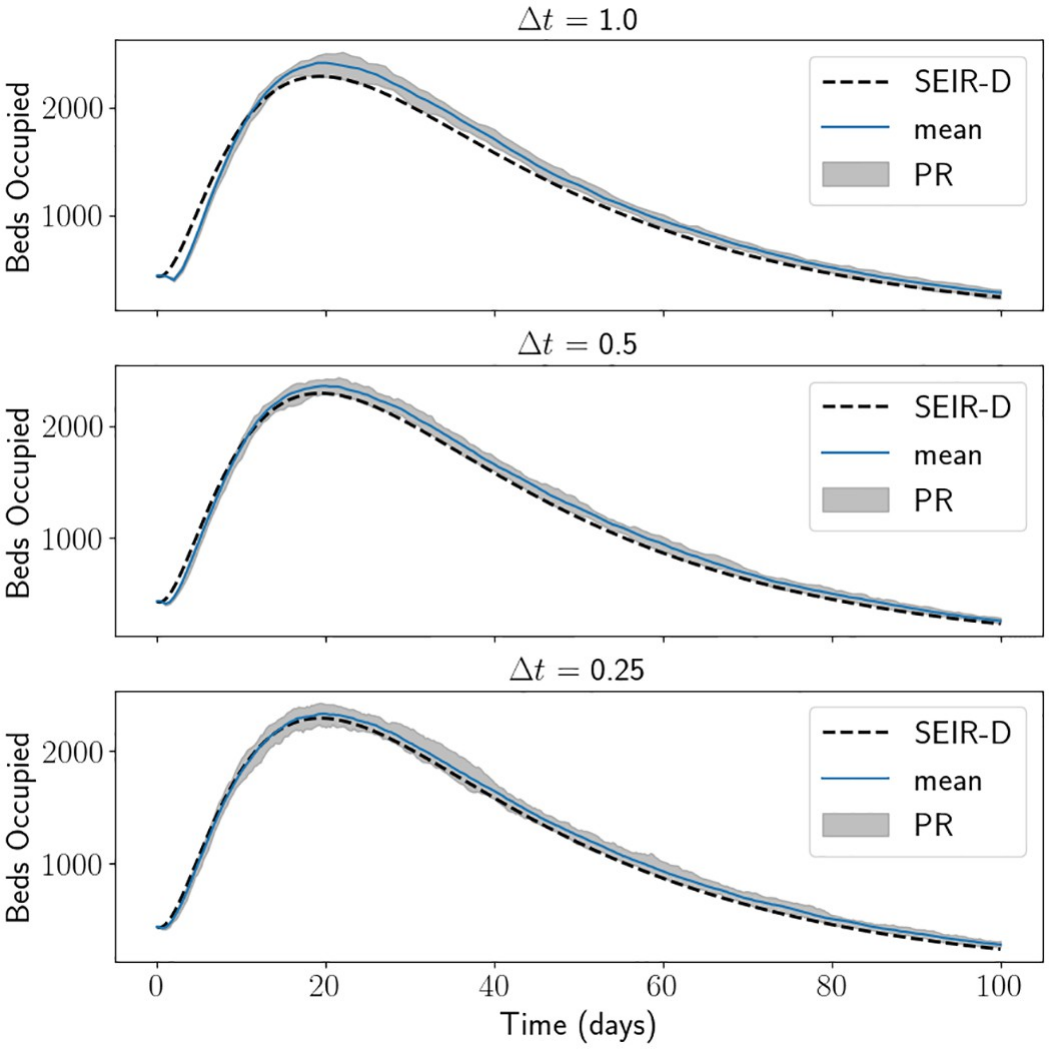
Beds occupied using the length of stay approach, using South East parameters and initial conditions with varied values for Δ*t*. The mean of the length of stay approach matches the SEIR-D results well for all values of Δ*t*. PR stands for percentile range.

Fig 7 is a comparison of the SEIR-D model with the length of stay approach with the correct term, using the South East parameters and initial conditions defined in Tables 1 and 2 respectively, taking several different values of Δ*t*. Here we have used 20 Monte Carlo simulations of the agent-based model. In practice, 20 Monte Carlo simulations are not enough to get an accurate measure of spread, however the algorithm is slow. This is due to the number of agents, approximately we are having to run conditions on around 10 million agents at each timestep. Unless one has extremely powerful computers, running multi-thread parallel computations, this approach is not appropriate for this population size. There are many techniques that can improve this simulation time, such as dynamic rescaling, but not to the speed of the branching process [50, 73, 74]. For this reason, for the remainder of the manuscript, we will use the branching process to generate our stochastic results. As expected, as Δ*t* decreases the mean of the length of stay approach starts to converge towards the output from the SEIR-D model. We again see that an agent-based approach overestimates the number of beds occupied, compared to the SEIR-D model, and possibly this could be due to the same reason as the branching process. The PR seems larger than the branching process, which is to be expected since there is a larger use of stochasticity in the length of stay model, due to event: transmission. However, it is not wise to draw too many conclusions about the spread due to the lack of Monte Carlo simulations.

## 3 Results: Interventions using hospital demand and capacity

For the remainder of this study we will use the parameters estimated in Table 1 but we will fix the initial conditions to represent the beginning of the epidemic. Namely, in Eqs (1) and (2) we set

$$\begin{array}{c} S_{0}=\lceil 0.999N\rceil ,\;\text{and}\;E_{0}=\lfloor 0.001N\rfloor , \end{array}$$ (28)

and for the remaining equations Eqs (3) to (9) we set the initial conditions equal to 0, where ⌈*x*⌉ represents the ceiling of *x* and ⌊*x*⌋ represents the floor of *x*. Fortunately, for the size of our populations, 0.1% of *N* is large enough to satisfy the mean-field approximation and thus we should see good agreement between the branching process and the SEIR-D model.

Using Eqs (1) to (9) we will model an intervention as a social distancing effect, by manipulating the average transmission rate *β*. We use two conditional statements, one which determines if the hospital capacity has been breached and one which determines when the demand is low enough, to dictate when an intervention is applied or lifted, and thus manipulate parameters during the simulation accordingly. This demonstrates a way to mathematically implement a scenario which could have resulted from a meeting with public health officials. We then pose an optimisation problem whereby we want to know when an intervention should be implemented to not breach capacity. Put together, these types of measurements are useful for population health management because, given an *R* number, one can provide an expectation of what could happen on average, and then set resource allocation appropriately. Throughout this study we will be measuring the “success” of an intervention by the percentage of individuals who have died throughout the simulation, in the sense that reducing this statistic means a more successful intervention.

For the branching process, we say that the outbreak is contained at a time *T* > 1 if *E*(*T*) + *U*(*T*) + *I*(*T*) = 0. This essentially says there are no more infections that can be spread throughout the community. For the SEIR-D model, we say that the outbreak is contained at a time *T* > 1 if *E*(*T*) + *U*(*T*) + *I*(*T*) < 1, $R_{t}<1$ and there is no ongoing intervention. This implies that herd immunity has been achieved and hence the system has reached its steady state. This description highlights one of the drawbacks of using deterministic equations over their stochastic counterparts, namely in the deterministic setting there is always “some amount” of the disease leftover in the community (i.e. 0 < *E*(*t*) + *I*(*t*) + *U*(*t*)). This can, mathematically, lead to another outbreak if the parameters are changed appropriately, which we would not expect to happen in reality.

We numerically approximate the solutions to the system Eqs (1) to (9) by using the SciPy implementation of the “lsoda” method, which is a combination of the Adams methods and the Backward Differentiation Formula (BDF) family of methods [75–77]. Given the multi-step approach of the ODE solver, each time we manipulate the parameters during a simulation we stop the current solver and start it again using initial conditions as the final values of the last solver. This bypasses numerical difficulties of having a discontinuous ODE system (with respect to the parameters).

### 3.1 Notation for an intervention

In the following sections we want to investigate how one can use hospital demand as a measurement for whether interventions are put into place. We aim to model the situation where an intervention is triggered when hospital capacity is almost full, and then lift the intervention when the hospital demand has reached an “opening” threshold of significantly lower patients. We denote the state of being in an intervention using the notation *ℓ*, namely if *ℓ* = 1 then we are in an intervention, otherwise we set *ℓ* = 0. We will introduce all the interventions in terms of the SEIR-D model and parameters for ease of exposition, but they work exactly the same for the branching process due to their similarities in expression. Using this, we describe the average transmission rate as

$$\begin{array}{c} \bar{\beta }\left(t;\ell \right)≔\left[\ell =1\right]\;\beta +\left[\ell =0\right]\;C_{R_{0}}\beta , \end{array}$$ (29)

where *β* is the average transmission rate associated to Table 1 for each region, $C_{R_{0}}$ is a scaling constant to give the initial value of $R_{0}$ wanted, and [⋅] are the Iverson brackets [78] defined as

$$\begin{array}{c} \left[P\right]≔\{\begin{array}{cc} 1 & \;\text{if}\;P\;\text{is}\;\text{true,}\\ 0 & \;\text{otherwise.} \end{array} \end{array}$$

We describe the intervention in the following recursive way

$$\begin{array}{c} \ell ≔\left[\ell =0\right]\left[H\left(t\right)>H_{u}\right]+\left[\ell =1\right]\left[H\left(t\right)>H_{l}\right], \end{array}$$ (30)

which is to say that the intervention is triggered at time *t* when the number of patients in hospital goes above an upper limit *H*_*u*_, and the intervention stays in place until the number of patients in hospital goes below a lower limit *H*_*l*_. Initially *ℓ* is set to 0. The values of *H*_*u*_ and *H*_*l*_ are regionally dependent since they depend on the maximum capacity of all the hospitals in a region. To enable comparisons between regions, we take *H*_*u*_ to be proportional of the total population, and *H*_*l*_ to be a proportion of *H*_*u*_.

We acknowledge that, in reality, the average transmission rate would not only go between two values. In fact, the recommended approach would be to estimate the transmission for each major policy change or each new dominant variant. We do not discuss this further in this study, but we recommend readers to the following studies and the references therein [79, 80].

### 3.2 The do-nothing approach

**Fig 8:**
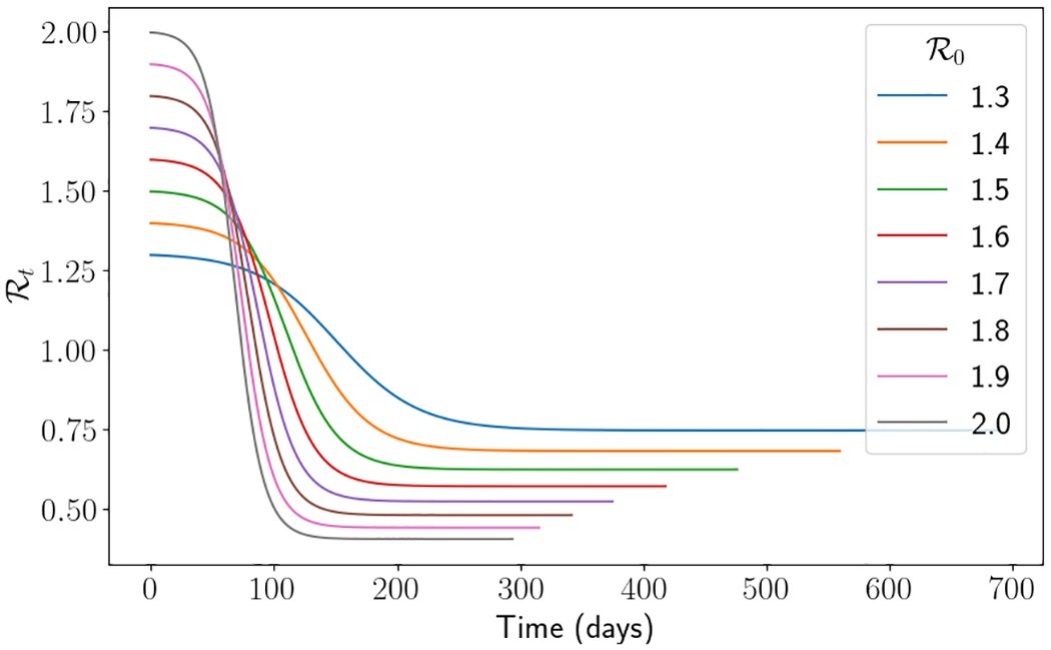
$R_{t}$ using the do-nothing approach with the England parameters.

**Fig 9:**
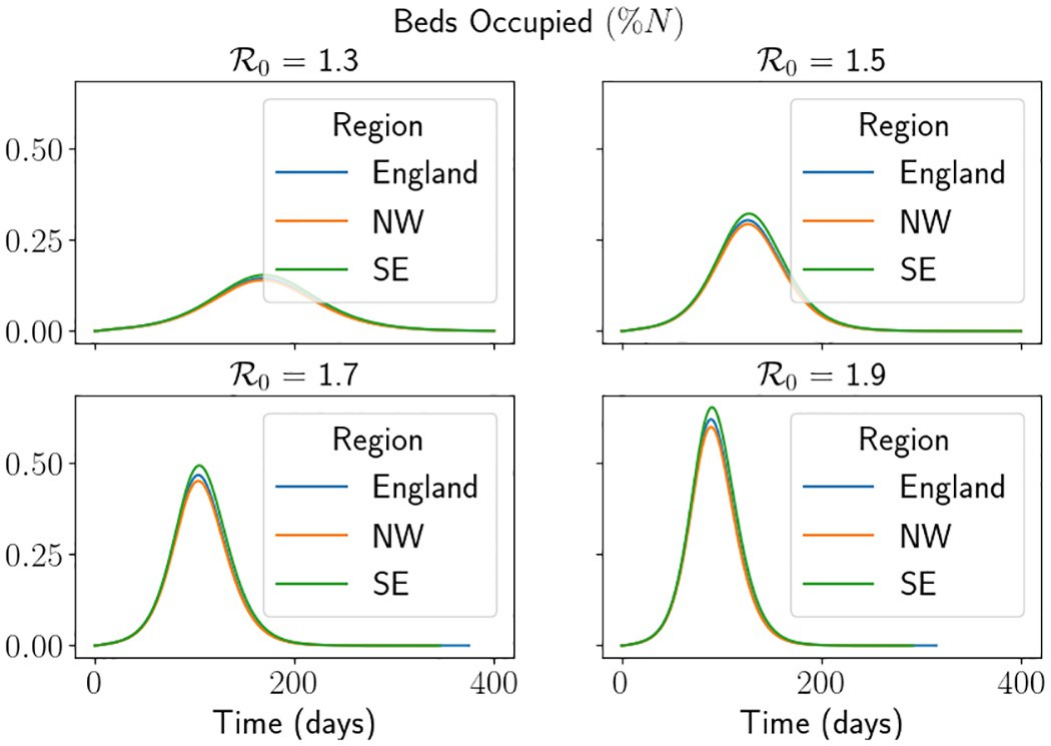
Percentage of beds occupied each day using the do-nothing approach for each region. We note that we have truncated the simulation to make visualisation easier. Here North West is abbreviated to NW and South East is abbreviated to SE.

This section demonstrates the do-nothing approach, which is simply to let the disease take its course, which we will use as a reference measure to see how the interventions are performing. For this, we take

$$\begin{array}{c} \bar{\beta }\left(t;\ell \right)≔C_{R_{0}}\beta , \end{array}$$

with *H*_*l*_ = 0 and *H*_*u*_ = ∞, i.e. we do not consider them at all. To get an idea of what a standard epidemic looks like using the SEIR-D framework, we present the following two figures. In Fig 8 we demonstrate the effective reproduction number $R_{t}$ of the simulation using the England parameters. This shows us that, when $R_{0}$ is larger, the actual epidemic is much shorter in length and reaches much smaller values of $R_{t}$. This description follows the notion that the larger the value of $R_{0}$, the more aggressive the disease is following the exponential growth of those who are infectious, as can be seen in Fig 8 by the steep decline in $R_{t}$. In other words, a small final value of $R_{t}$ means that more individuals were infected, since it is proportional to *S*(*t*). In Fig 9 we demonstrate a comparison of the percentage of beds occupied for each of the regions and for some values of $R_{0}$. In Fig 9 we have truncated the simulation to make the visualisation easier. Interestingly, between regions, the day of the peak does not seem to change even though the hospitalisation parameters are all quite varied. We suspect that this is somehow related to the initial conditions and the fact that $R_{0}$ is not effected by the parameters that describe hospitalisations.

Although it is clear that we are using the same parameters in both the SEIR-D model and the branching model, because we are scaling the average transmission rate we need to be clear on what that means in the agent-based approach. As mentioned before, we have the relation *β* ≔ *Ca*, thus to generate $C_{R_{0}}$ we can scale either *C* or *a*. Thus, an increase in *β* can simply be interpreted as more individuals meeting each other and spreading the disease (increase in *C*), or the average probability of a successful transmission has increased due to a new variant (increase in *a*). In Table 3 we demonstrate that scaling *C* or scaling *a* makes very little difference to the mean, as to be expected, but also does not affect the spread. From here onwards, we will fix *C* = 9 and let *a* vary when $R_{0}$ varies.

**Table 3:** The do-nothing approach comparing the SEIR-D model with the branching process using the South East parameters. Displaying the number of dead individuals (as a percentage of the population), changing the average number of contacts *C* or the average transmission rate *a*, taking Δ*t* = 0.25. BM stands for the mean of the results from branching model and PR is the percentile range.

	Fix *a* = 0.0303		Fix *C* = 9	
$R_{0}$	Mean	(PR)	Mean	(PR)
1.3	1.032%	(1.024%, 1.042%)	1.033%	(1.024%, 1.042%)
1.4	1.244%	(1.234%, 1.255%)	1.244%	(1.235%, 1.254%)
1.5	1.418%	(1.408%, 1.427%)	1.418%	(1.408%, 1.428%)
1.6	1.560%	(1.550%, 1.570%)	1.560%	(1.551%, 1.570%)
1.7	1.679%	(1.669%, 1.689%)	1.680%	(1.669%, 1.689%)
1.8	1.779%	(1.768%, 1.789%)	1.779%	(1.769%, 1.790%)
1.9	1.864%	(1.853%, 1.874%)	1.864%	(1.853%, 1.874%)
2.0	1.935%	(1.924%, 1.946%)	1.936%	(1.925%, 1.946%)

In Table 4 we measure the maximum number of beds occupied in hospitals as a percentage of the total population and the day in the simulation when it is reached using the South East parameters. Namely, we measure

$$\begin{array}{cc} M_{beds} & ≔\underset{t\in [0,T]}{\text{max}}\frac{H(t)}{N},\\ M_{day} & ≔\tau ,\;\;\text{such}\;\text{that}\;\;M_{beds}=\frac{H(\tau )}{N}. \end{array}$$

**Table 4:** The do-nothing approach comparing the SEIR-D model with the branching process using the South East parameters. Displaying the maximum number of beds occupied (as a percentage of the population) and what day the simulation reaches that maximum, taking Δ*t* = 0.25. BM stands for the mean of the results from branching model and PR is the percentile range.

	Max beds occupied (%*N*)			Peak of beds occupied (day)		
$R_{0}$	SEIR-D	BM	(PR)	SEIR-D	BM	(PR)
1.3	0.154%	0.155%	(0.151%, 0.158%)	169.6	169.9	(162.8, 178.0)
1.4	0.236%	0.237%	(0.233%, 0.241%)	144.8	145.4	(140.7, 150.8)
1.5	0.322%	0.323%	(0.318%, 0.328%)	127.5	128.1	(124.5, 131.5)
1.6	0.408%	0.410%	(0.405%, 0.415%)	114.7	115.2	(112.2, 118.2)
1.7	0.492%	0.495%	(0.489%, 0.500%)	104.8	105.4	(103.0, 107.8)
1.8	0.574%	0.576%	(0.570%, 0.583%)	96.9	97.5	(95.5, 99.5)
1.9	0.652%	0.655%	(0.649%, 0.661%)	90.4	91.1	(89.5, 93.0)
2.0	0.725%	0.729%	(0.723%, 0.736%)	85	85.7	(84.2, 87.2)

**Fig 10:**
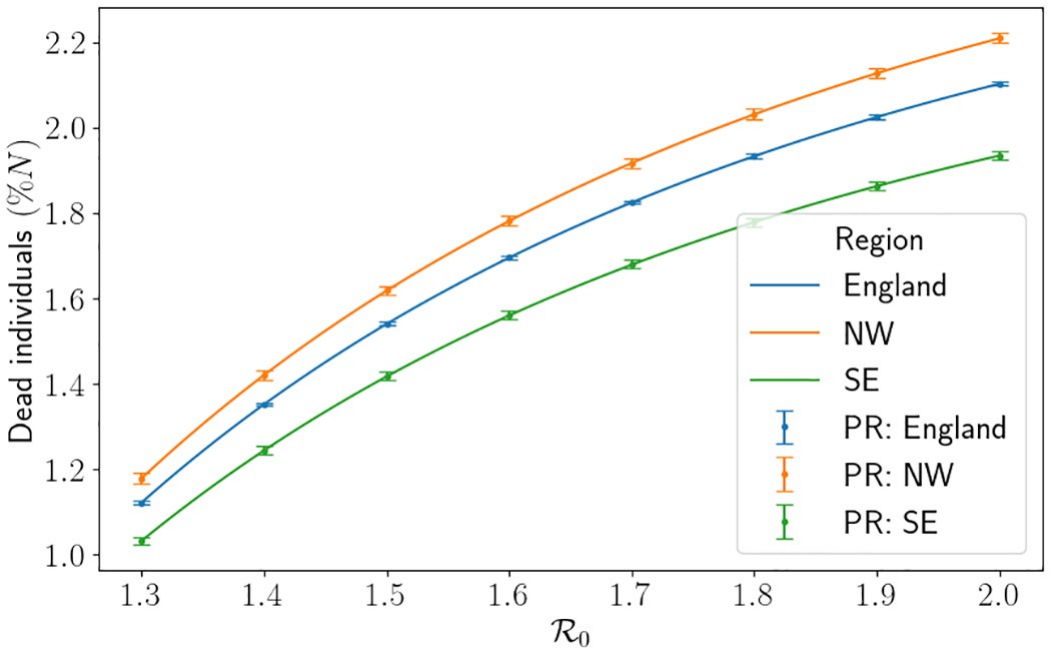
The do-nothing approach comparing the SEIR-D model with the branching process for all regions. Depicting the number of dead individuals (as a percentage of the population) against $R_{0}$, taking Δ*t* = 0.25. PR is the percentile range from the branching model. Here North West is abbreviated to NW and South East is abbreviated to SE.

The results of the simulations using the other regional parameters can be found in S1 Appendix. In Fig 10 we depict the percentage of dead individuals at the end of the outbreak for each region. Namely, we measure

$$\begin{array}{c} M_{d}≔\frac{D_{U}\left(T\right)+D_{H}\left(T\right)}{N}. \end{array}$$

We demonstrate the results using the SEIR-D model and also the mean and PR of the branching process, using 1000 Monte Carlo simulations with Δ*t* = 0.25. As intuitively expected, as $R_{0}$ increases the percentage of the highest demand in the hospital increases, the day of that peak is sooner and the percentage of dead individuals increases. We see that the percentage of dead individuals is highest in the North West, then in England and then in the South East. This is due to the ratio $\gamma_{H}\mu_{H}^{-1}$ being largest in the North West which implies that there are less discharges for every one death. On the flip side, we see that the percentage of maximum beds occupied is worse in the South East, followed by England and then the North West. This is due to the average removal rate from hospitals *γ*_*H*_ + *μ*_*H*_ being the largest in the South East. We have good agreement between the SEIR-D model and the branching model, but as $R_{0}$ increases the differences between the outputs become larger. Considering the hospital bed demand, as $R_{0}$ increases, the interpercentile range also increases, going from 7% difference (approximately 634 beds occupied in the South East) to 13% difference (approximately 1244 beds occupied in the South East). We suspect this is due to higher variance in the overall number of successful infections. Interestingly, the interpercentile range for the peak of the demand actually decreases when $R_{0}$ increases. We suspect this is due to the length of the simulation decreasing. One can also computationally see the effect of population size on the PR in Fig 10 since the PRs for England are much smaller than the corresponding ones for the North West.

### 3.3 Fixing the limit on demand

**Fig 11:**
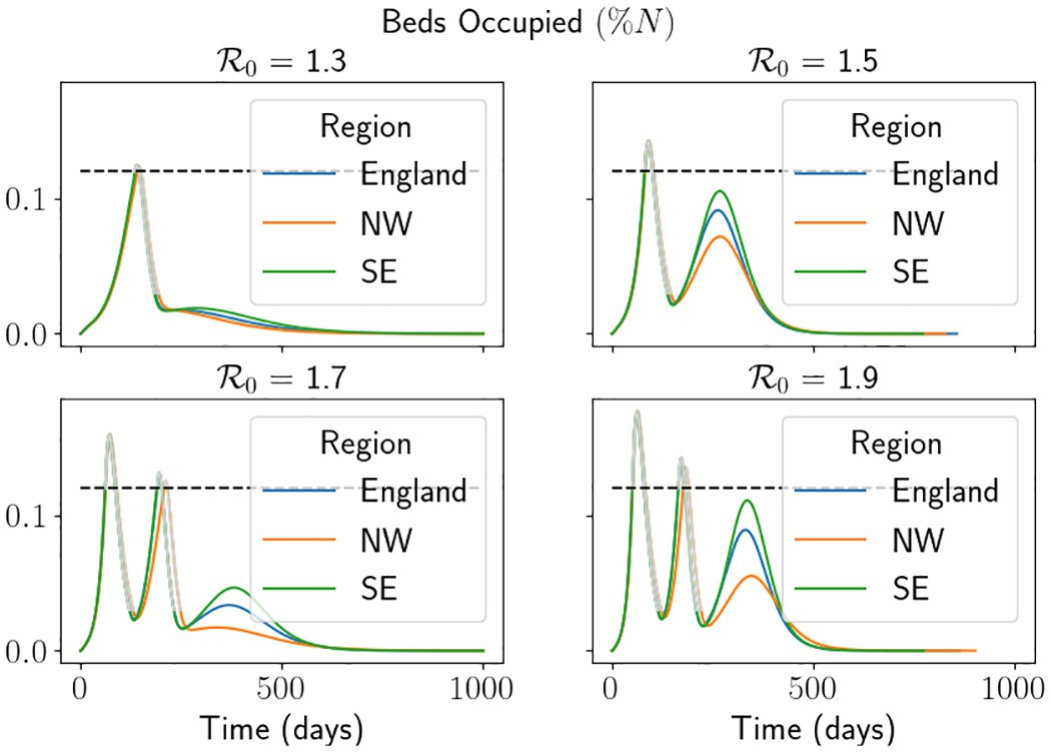
Percentage of beds occupied each day using the hospital capacity intervention scenario for each region. We fix *H*_*l*_ ≔ 0.25*H*_*u*_. The grey lines represent the times when the simulation is in an intervention, and the black dashed line represents *H*_*u*_*N*^−1^. We note that we have truncated the simulation to make visualisation easier. Here North West is abbreviated to NW and South East is abbreviated to SE.

In this section, we will demonstrate the demand and capacity intervention and compare the results against the previous section. Here we will vary $R_{0}$ whilst fixing *H*_*l*_ to understand the role $R_{0}$ has to play. We will fix *H*_*l*_ ≔ 0.25*H*_*u*_ or *H*_*l*_ ≔ 0.5*H*_*u*_. Given the maximum hospital demand in Table 4, and equivalent tables in S1 Appendix, we fix *H*_*u*_ ≔ 0.0012*N* to guarantee at least one intervention for all values of $R_{0}$ chosen in each region. We restrict most of the figures in the following sections to depict the results for the South East region so the figures are not a visual burden. The results for the other regions are in S1 Appendix. One can see in Figs 10 and 11 that the results for each region follow a similar trend.

**Fig 12:**
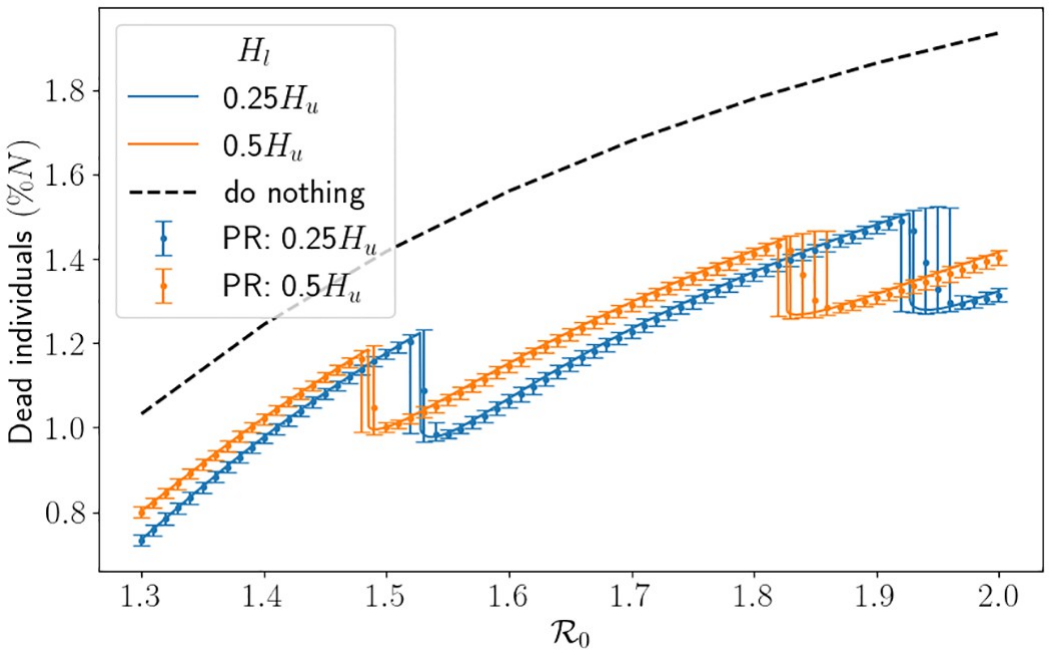
Percentage of dead individuals corresponding to an $R_{0}$ value using the hospital capacity intervention approach and the South East parameters. Here we have fixed *H*_*l*_ ≔ 0.25*H*_*u*_ or *H*_*l*_ ≔ 0.5*H*_*u*_, and set Δ*t* = 0.5. The thick line represents the results from the SEIR-D model, and the error bars depict the result from the branching model. The black dashed line depicts the associated percentage of dead individuals using the do-nothing approach. PR is the percentile range from the branching model.

**Fig 13:**
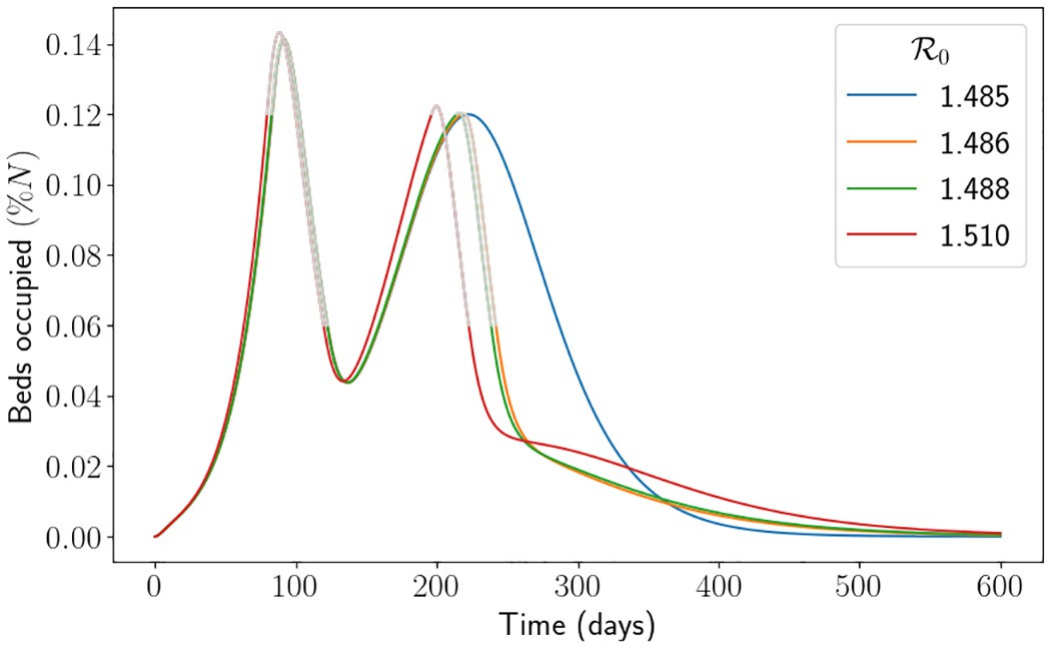
Percentage of patients in hospitals per day using the hospital capacity intervention approach and the South East parameters. Here we have fixed *H*_*l*_ ≔ 0.5*H*_*u*_. The grey lines represent the times when the simulation is in an intervention. We note that we have truncated the simulation to make visualisation easier.

**Fig 14:**
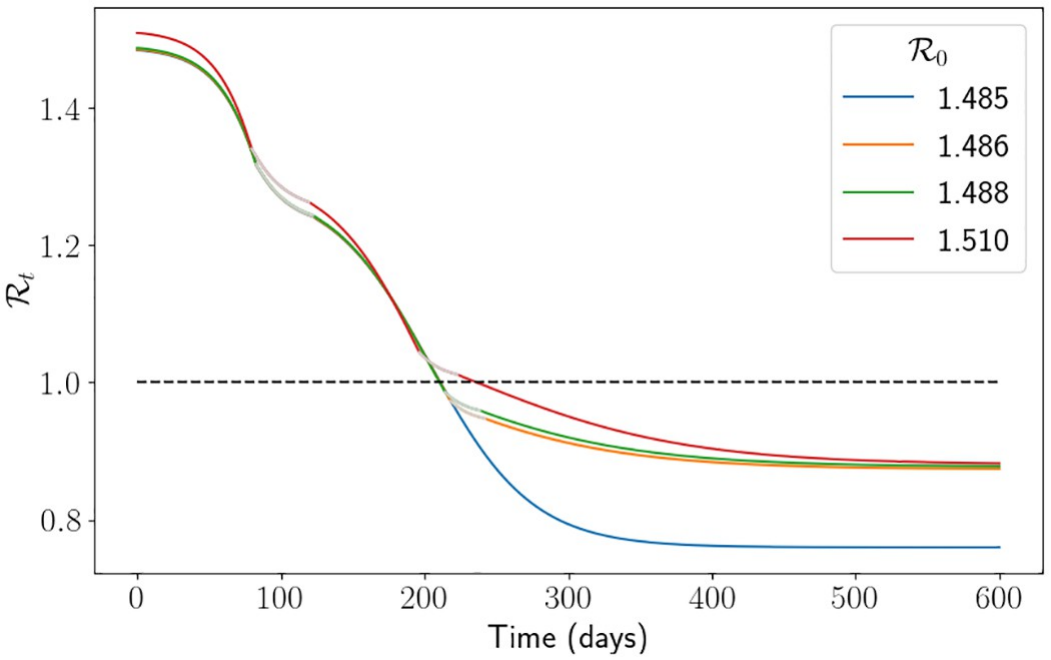
$R_{t}$ per day using the hospital capacity intervention approach and the South East parameters. Here we have fixed *H*_*l*_ ≔ 0.5*H*_*u*_. The grey lines represent the times when the simulation is in an intervention. The black dashed line represents herd immunity $R_{t}=1$. We note that we have truncated the simulation to make visualisation easier.

In Fig 12 we depict the percentage of dead individuals using the South East parameters. One can easily see that the interventions have a strong impact on the proportion of dead individuals, particularly when $R_{0}$ is large. In this figure, each drop signifies an extra intervention has been initiated (the intervention at around $R_{0}=1.5$ is the second intervention of the simulation). It is to be expected that there is a drop in deaths when a new intervention is initiated because it heavily affects the transmission rate, and thus the disease does not reach individuals who would have died upon being infected. What is not necessarily intuitive is that the proportion of deaths continues to decrease for a short while past the first value of $R_{0}$ that triggers an extra intervention (this is easiest to see at the second drop for *H*_*l*_ = 0.25*H*_*u*_). We suspect this is due to the value of $R_{t}$ when the intervention is lifted. To demonstrate this, setting *H*_*l*_ = 0.5, we carefully picked four values of $R_{0}$ in Figs 13 and 14. $R_{0}=1.485$ is just before the drop in Fig 12, $R_{0}=1.486$ and $R_{0}=1.488$ are just after the drop and $R_{0}=1.51$ is quite far after the drop. What we expect is that, in Fig 13, the curve associated to $R_{0}=1.488$ is above the curve associated to $R_{0}=1.486$ since the infection rate is larger, however we see that when the second intervention is lifted the curve associated to $R_{0}=1.488$ is in fact lower. In Fig 14 we see that the same is happening, and so the value of $R_{t}$ is higher for the curve associated to $R_{0}=1.488$ (which means that its number of susceptibles is higher). However, since $R_{t}<1$ the system is in the state of herd-immunity, and so the number of new infections is decreasing. Moreover, the shallow decrease of $R_{t}$ implies there are very little new infections. All in all, with slightly more susceptibles leftover, this implies that less infections overall have occurred, and thus why the number of deaths is lower. We also see some uncertainty around the impact of the interventions, with the spread of each simulation being significantly larger around the $R_{0}$ value which causes an intervention. The branching process manages to capture the first drop well on average, but it starts to struggle to capture the second drop and instead smoothens it out. Understanding analytically when the interventions are initiated and the resulting $R_{0}$ which minimises the number of deaths is left for future research. Furthermore, computationally finding the optimal values of *H*_*u*_ and *H*_*l*_ to minimise the number of deaths given an $R_{0}$ is also left for future research. Nevertheless, this interplay between interventions, parameter values and herd immunity is difficult to analyse and demonstrates the benefit of mathematically modelling for forecasting.

### 3.4 Varying lower limit of demand

In this section, we will be experimenting with the limit of demand that signals for a lifting of an intervention. Here we will fix $R_{0}=1.5$ and vary *H*_*l*_ to understand the role *H*_*l*_ has to play.

**Fig 15:**
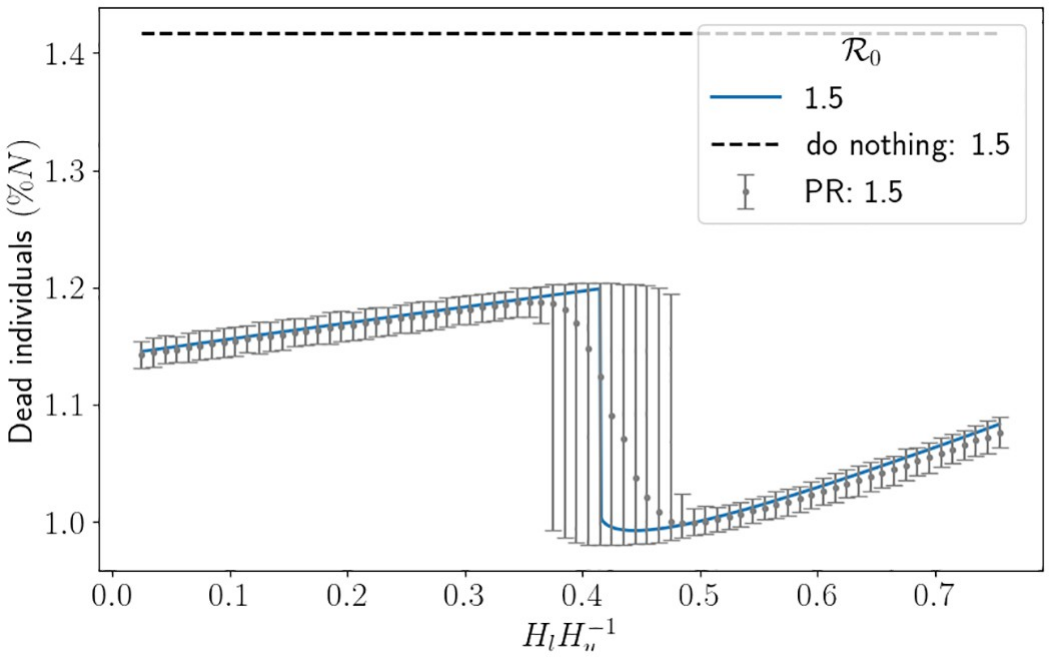
Percentage of dead individuals corresponding to an $R_{0}$ value using the hospital capacity intervention approach and the South East parameters. The thick line represents the results from the SEIR-D model, and the error bars depict the result from the branching model. Here we have fixed $R_{0}≔1.5$ and set Δ*t* = 0.5. The black dashed line depicts the associated percentage of dead individuals using the do-nothing approach. PR is the percentile range from the branching model.

**Fig 16:**
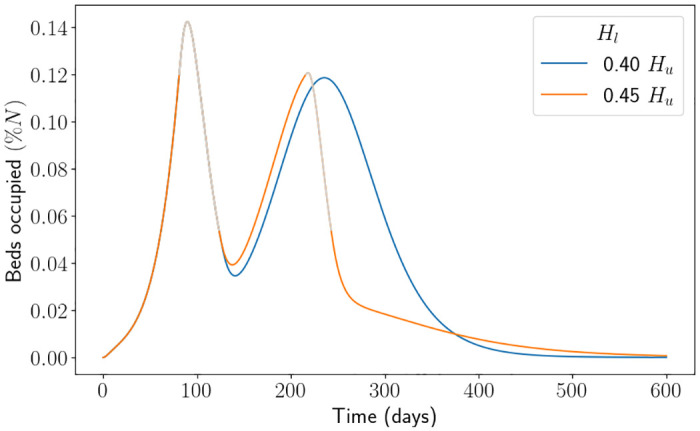
Percentage of patients in hospitals per day using the hospital capacity intervention approach and the South East parameters. Here we have fixed $R_{0}=1.5$. The grey lines represent the times when the simulation is in an intervention. We note that we have truncated the simulation to make visualisation easier.

In Fig 15 we depict the percentage of dead individuals using the South East parameters. We see that again the intervention has dramatically decreased the percentage of deaths. Interestingly, changing the threshold *H*_*l*_ does not have much impact on the percentage of deaths, but rather affects the speed at which interventions are lifted. One sees the impact of an extra intervention being initiated, which can also be seen in Fig 16. The uncertainty around the intervention initiation is surprisingly large. Doubling the number of Monte Carlo simulations and halving Δ*t* had no effect on reducing the uncertainty which we found quite interesting.

The outcomes of our study so far imply that the timing and the lengths of interventions are extremely important. Getting closer to herd immunity when ending an intervention has the potential to save a huge number of lives. However, accurately calculating $R_{t}$ is in general very challenging which leaves the process of timing for herd immunity difficult. Although the percentage of total deaths decreases with an added intervention, the length of most of the interventions is large due to the criteria set, and so the simulation is prolonged significantly. This is mainly due to the fact that the average hospitalisation period is large and that the scenario we are simulating means that intervention will be in place until hospitals go from full capacity to between 2.5% and 75% capacity. Fortunately, as the maximum demand increases, the percentage of total deaths does not increase dramatically, and the length of interventions decreases from the best part of 4 months to under 1 month. In reality, as the outbreak progresses, one would expect the average hospitalisation period to decrease, since awareness of the disease and treatment gets better, as well as an increase in resources and the development of vaccines. This final point is important as it means realistic interventions can be implemented as circuit breakers and still maintain a large decrease in the number of total deaths.

### 3.5 Optimisation of upper limit of demand

In this section we calculate what the highest demand threshold *H*_*u*_ is so that hospitals do not go over their maximum capacity *H*_*max*_, which we fix at *H*_*max*_ ≔ 0.0012*N*. In particular, this approach can be used as an early warning system by outlining when interventions need to be enforced to maintain manageable demand. It can complement scenario-based forecasting approaches by giving a range of indicators of when to open up further capacity in hospitals or introduce an intervention which can be tracked against with incoming data daily. Ultimately, in practice, healthcare systems will want to utilise their capacity appropriately whilst not impacting the general public with an intervention, and so optimising the difference between the resource capacity and the maximum number of patients per parameter set is important.

In this section, we only consider the situation of finding *H*_*u*_ using one intervention, so Eq (30) becomes

$$\begin{array}{c} \ell ≔\left[\ell =0\right][H\left(t\right)>H_{u}], \end{array}$$

with Eq (29) the same. We consider one intervention here because it can be reasoned that a second spike in demand is essentially the same as the first spike but with a smaller $R_{0}$.

**Fig 17:**
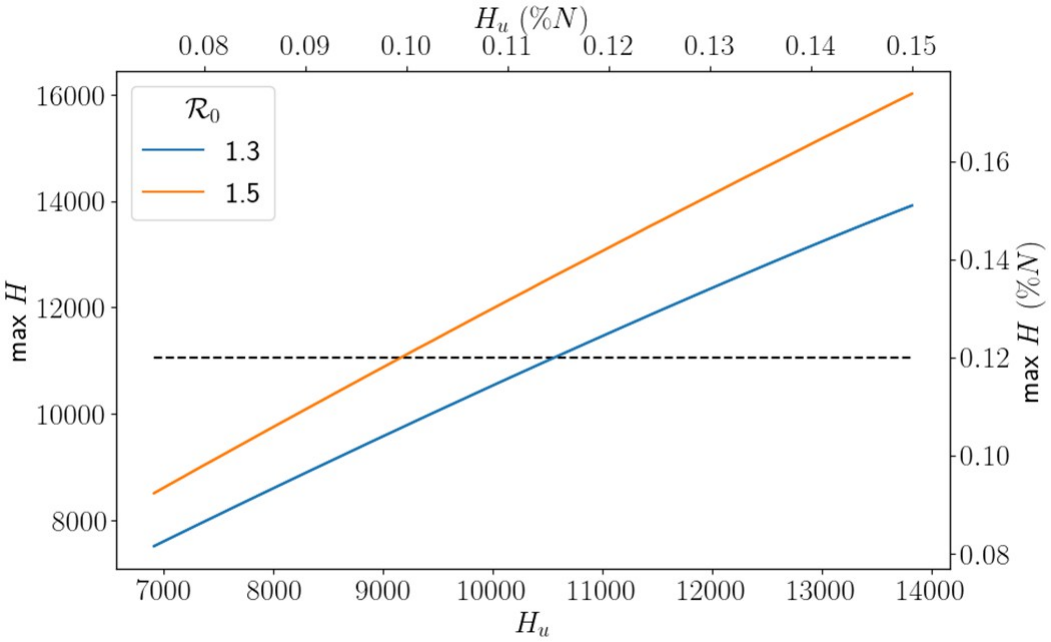
Comparison of *H*_*u*_ against the max *H* for different values of $R_{0}$ using the SEIR-D model and the South East parameters. The black dashed line represents *H*_*max*_.

**Fig 18:**
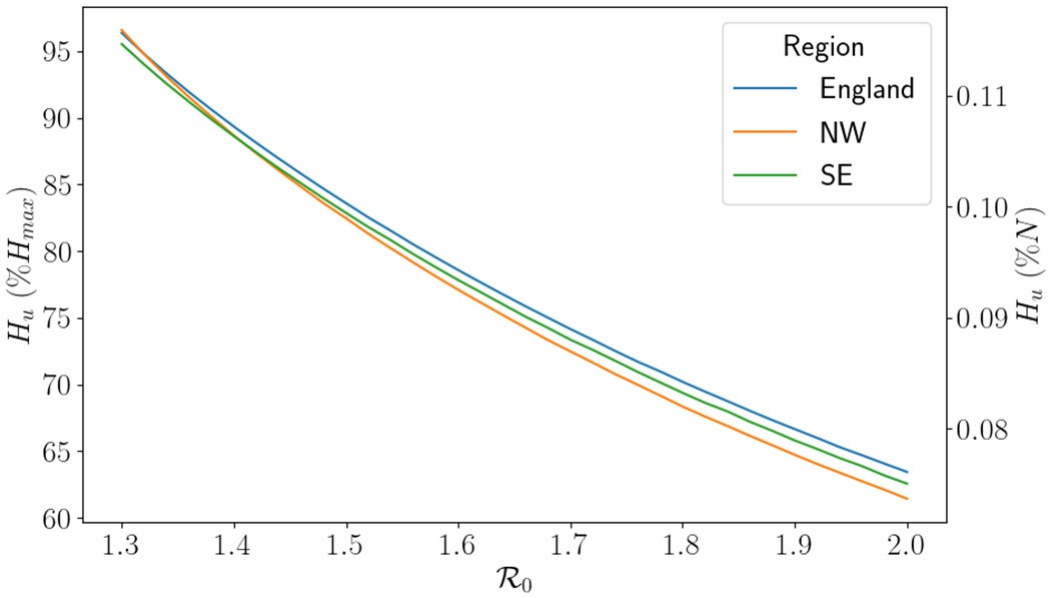
The values of *H*_*u*_ as a percentage of *H*_*max*_ corresponding to the roots of L for different values of $R_{0}$. Here North West is abbreviated to NW and South East is abbreviated to SE.

For a specific value of $R_{0}$, we look to find the root of the function

$$\begin{array}{c} L\left(H_{u};H,H_{max}\right)≔\underset{t>0}{\text{max}}\;H\left(t\right)-H_{max}, \end{array}$$

where we note that *H* depends on *H*_*u*_ due to *ℓ* and $\bar{\beta }$. Since *H* is continuous, the function L is continuous and will always have a root provided $R_{0}$ is chosen high enough, see Fig 17. A value of $R_{0}$ chosen too small means that an intervention is not initiated, and so there are no capacity issues. The results of the optimisation problem are presented in Fig 18. This figure should be read in the following way: if we know $R_{0}$, then we look to determine what percentage of our capacity is the threshold for demand before an intervention is initiated. As expected, as $R_{0}$ increases the percentage of capacity needed for an intervention decreases.

### 3.6 Uncertainty quantification of breaches

Now that we have the limits *H*_*u*_ which, on average, guarantee that hospital capacity is not breached, we want to observe how stochasticity affects these results. In particular, we want to calculate the proportion of the Monte Carlo simulations that still result in a breached capacity and how the number of realisations decreases when the threshold for an intervention is reduced. This investigation can act as some sort of buffer for hospital management to understand how close to the threshold *H*_*u*_ they can get before having to make resource changes. We note here that the agent-based approach tends to overestimate its deterministic counterpart which means that the results from the branching model will be skewed towards a breach in the threshold. In this investigation we measure three metrics, the proportion of Monte Carlo realisations that go over *H*_*max*_, the average maximum amount the realisations that go above *H*_*max*_ (to give an idea of how severely the threshold is breached), and the average maximum difference of the simulated hospital capacity *H* from *H*_*max*_ across all realisations. Namely, we measure

$$\begin{array}{cc} M_{max} & ≔\frac{1}{M_{b}}\frac{1}{H_{max}}\sum_{m=1}^{M}\left[L_{m}>0\right]\;L_{m},\\ M_{diff} & ≔\frac{1}{M}\frac{1}{H_{max}}\sum_{m=1}^{M}L_{m}, \end{array}$$

where

$$\begin{array}{c} L_{m}≔L\left(H_{u};H_{m},H_{max}\right), \end{array}$$

*H*_*m*_ denotes a Monte Carlo realisation of *H*, *M* denotes the number of Monte Carlo simulations ran, and *M*_*b*_ is the number of Monte Carlo simulations that resulted in a breach. We investigate several different values of $R_{0}$ and their associated value of *H*_*u*_ from Fig 18. Moreover, we investigate how *H*_*u*_ impacts these metric but varying it to find what proportion results in zero breaches and what proportion results in 100% breaches, of 5000 Monte Carlo trials. In the following simulations, we have taken Δ*t* = 0.25.

**Fig 19:**
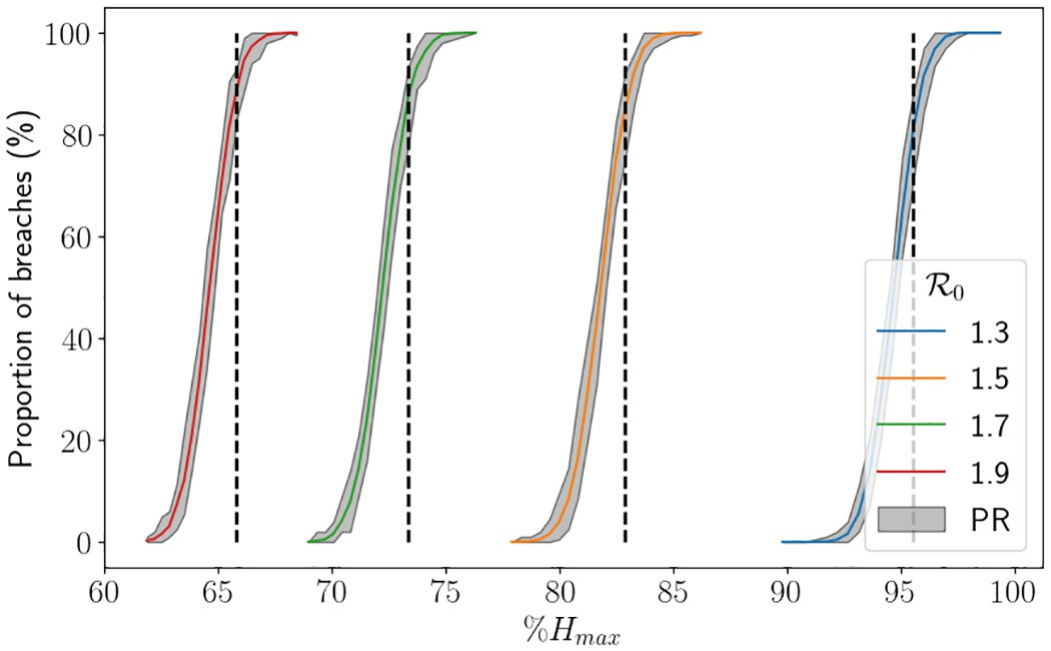
Proportion of breaches against different proportions of *H*_*u*_ associated to the value of $R_{0}$, which have then been converted into a proportion of *H*_*max*_, using the hospital capacity intervention approach (finishing after one intervention) and the South East parameters, with Δ*t* = 0.25. PR stands for the percentile range from the branching model and the thick lines represent the mean from the branching model. The black dashed line represents the value of *H*_*u*_ associated to $R_{0}$ found in Fig 18.

**Fig 20:**
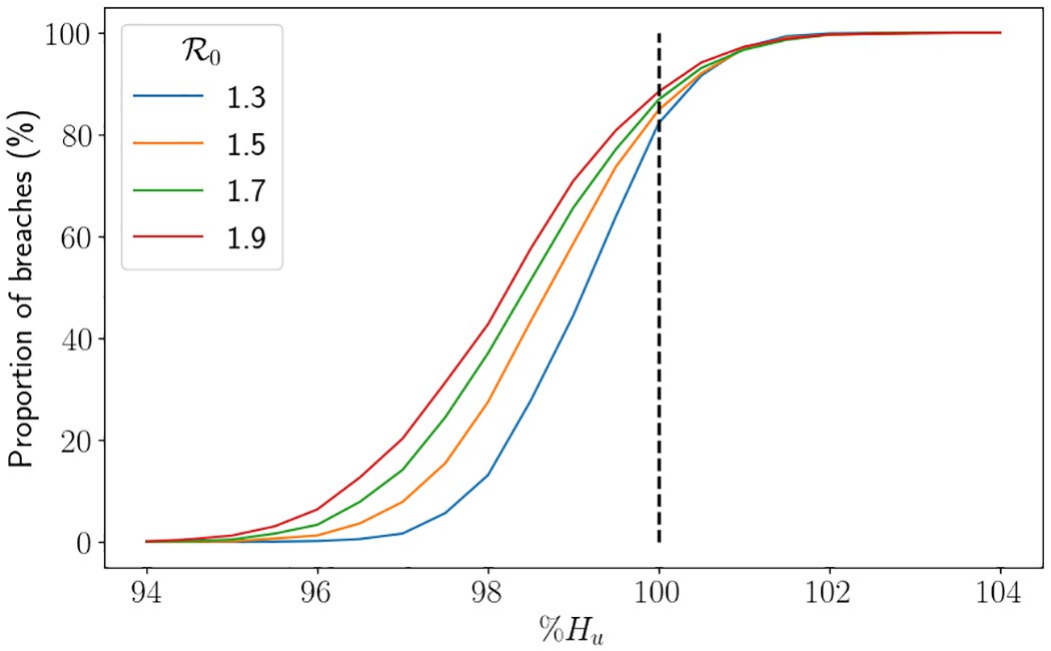
Proportion of breaches against different proportions of *H*_*u*_ associated to the value of $R_{0}$ using the hospital capacity intervention approach (finishing after one intervention) and the South East parameters, with Δ*t* = 0.25. PR stands for the percentile range from the branching model and the thick lines represent the mean from the branching model.

**Fig 21:**
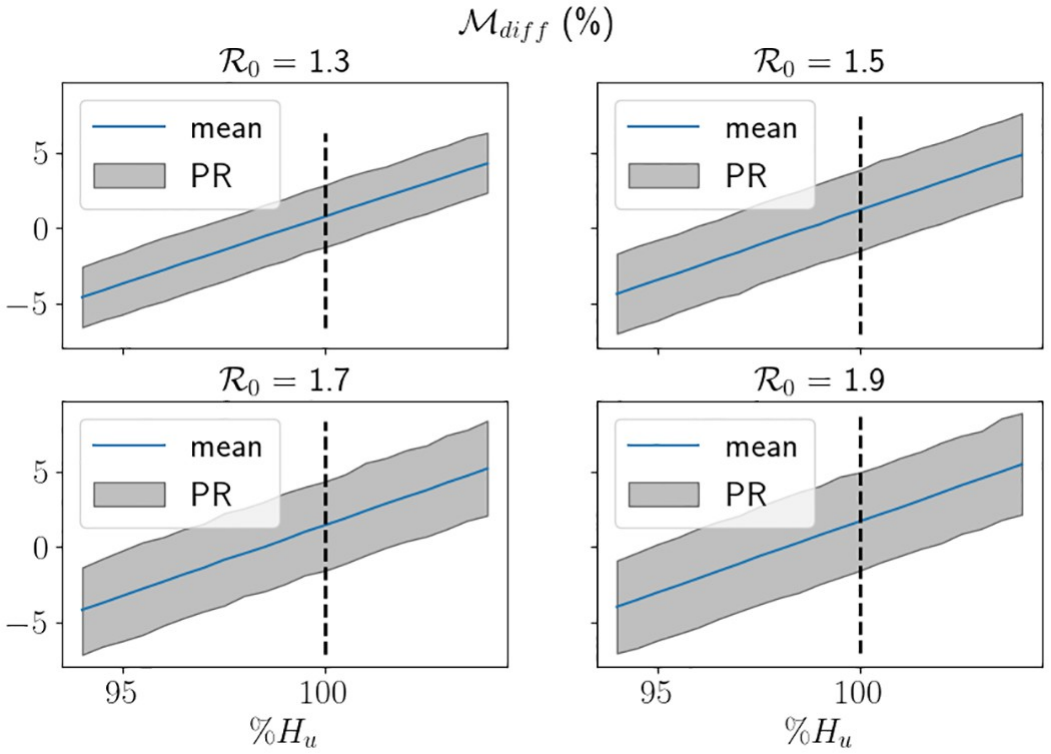
$M_{diff}$ against different proportions of *H*_*u*_ associated to the value of $R_{0}$ using the hospital capacity intervention approach (finishing after one intervention) and the South East parameters, with Δ*t* = 0.25. PR stands for the percentile range from the branching model and the thick line represent the mean from the branching model.

**Fig 22:**
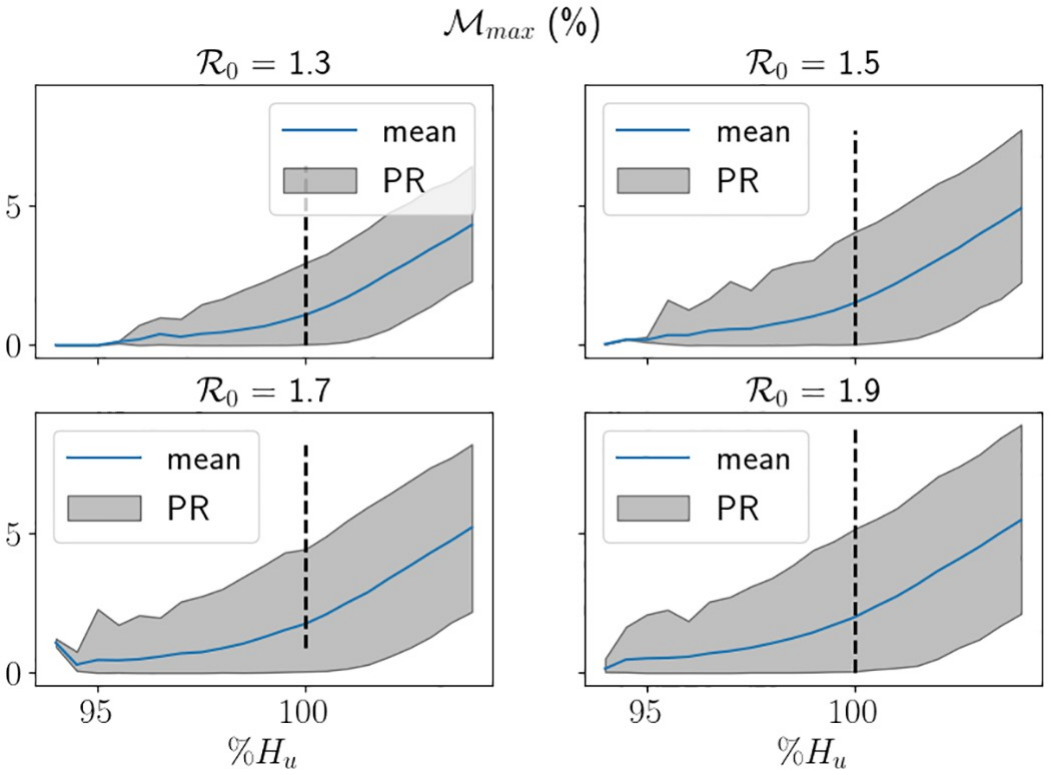
$M_{max}$ against different proportions of *H*_*u*_ associated to the value of $R_{0}$ using the hospital capacity intervention approach (finishing after one intervention) and the South East parameters, with Δ*t* = 0.25. PR stands for the percentile range from the branching model and the thick line represent the mean from the branching model.

In Fig 19 we display the proportion of breaches using a range of *H*_*u*_ values for different $R_{0}$ values, whereby the *x*–axis is the value of *H*_*u*_ as a percentage of *H*_*max*_. For this figure, in order to provide the percentile range, we split the 5000 trials into 50 sets of 100 simulations. In the figure, the black dashed lines represent the associated value of *H*_*u*_ from Fig 18, which roughly corresponds to between 80%-85% of simulations ending as a breach. Although it is tough to see, the uncertainty is larger for larger values of $R_{0}$, which is to be expected. We also see that the range of percentages of *H*_*max*_ representing 0% breaches to 100% breaches is larger for smaller values of $R_{0}$, which is due to the larger value of *H*_*u*_. Indeed, in Fig 20, we see that in fact the equivalent range of *H*_*u*_ values is smaller for smaller values $R_{0}$, which is intuitive. In Fig 21 we depict $M_{diff}$. For the values of *H*_*u*_ chosen, the difference ranges between max *H* being around 5% less than *H*_*max*_ to being around 5% greater than *H*_*max*_, where 5% is approximately 3400 beds. We see quite clearly that the percentile range is significantly larger for larger values of $R_{0}$, which is consistent with our findings throughout this study. It was, however, surprising to see that the percentage differences on average were very similar in values for all $R_{0}$ considered. In Fig 22 we depict $M_{max}$. Again, we see the uncertainty is larger for larger values of $R_{0}$, and that is takes higher demand for smaller values of $R_{0}$ to start breaching consistently and by large amounts.

## 4 Discussion: Limitations of the modelling approach and their mitigation

In this study, we assumed that the average probability of going to hospital is the same throughout the different regions due to problems with parameter estimation and the initial conditions. We speculate that the issue of estimating the initial conditions without keeping *p* fixed is solvable by reformulating the nonlinear initial value problem into a non-linear boundary value problem, where the data is used directly in the model as boundary points rather than an attribute of the fitting process. We have so far demonstrated how the boundary value problem would be conceived for a simple SIR model and prove existence and uniqueness of the problem for fixed parameters. In the future we look at parameter identifiability, parameter estimation and efficient numerical algorithms using this method [56]. The standard methods of choice to solve these types of boundary value problems are the shooting method, nonlinear least squares of the equivalent initial value problem, and numerical continuation [81–84]. It is not clear which method is the most appropriate for the solution to the nonlinear boundary value problems that can be derived from models used in epidemiology.

Apart from issues of parameter estimation, we have made a few assumptions about the agents and interventions. Firstly, in reality it is somewhat unrealistic that transmission would immediately revert to normal amounts after an intervention [85]. There would likely be a brief moment of time whereby transmission is reduced and then a knee jerk reaction back to normality, or there would be a gradual increase in contacts due to further interventions, like the tiered system. Another oversight of the work here is that the county is not homogeneous with respect to age. Different age-groups have different social structures, responsibilities—such as working, school life, or family, and different responses to a COVID-19 infection. In this work, we have not explored the impact of inhomogeneity within the population on hospital demand and capacity, in principle due to the lack of publicly available data. In general, the intervention we impose on our system is a total lockdown of all ages, similar to the national lockdown during the first wave, however utilising age-groups within the model will allow for dedicated forecasting into the effect some social events, like schools opening or returning to offices, will have on interventions [86–88].

From an operational perspective, another aspect to consider with this study is that we are combining the capacity of all the hospitals in a region, due to modelling constraints and data access. This means that we are assuming hospitals can move patients to other hospitals throughout each region in response to the bed capacity of each individual hospital, which is not a realistic assumption. In order to overcome this issue, we would have to be able to model each trust individually. Other than data availability issues, it might be difficult to understand the catchment area corresponding to each trust without having insider knowledge, which means getting the total population size and death data difficult. However, the Office for Health Improvement and Disparities in the UK conducted some research demonstrating the complexity of catchment areas and regional borders [89]. Within hospitals, we have also overlooked the potential for nosocomial outbreaks, whereby the probability of an outbreak increases with a larger number of infectious patients, and general public compliance and attitude. This would involve an *H* term in the expression for λ(*t*), with a coefficient that dampens its addition to the average infection rate. In order to capture this in the data, one would need to be testing patients daily and then using some criteria on their length of stay and when they became positive to deduce a nosocomial infection.

On a similar note, the interventions we presented are mainly interpreted as non-pharmaceutical interventions. The addition of vaccinations and/or drugs adds on another layer of complexity that would be interesting to study. Particularly because vaccinations are typically pivotal in the role to combat epidemics and stop resurgences and outbreaks in the future [90–94]. Moreover, COVID-19 vaccination efficacy and rates are published and so these could easily be incorporated into our model. Mathematically, the addition of a vaccine into the model has been undertaken in previous works of similar nature [93, 95]. Both the agent-based models and the equation-based models have their own paradigms of ways to model vaccinations. The sterilizing vaccine approach assumes that a proportion of those receiving vaccines gain (some form of) immunity whilst the others do not. This style of approach lends itself well to agent-based approaches due to the application of a criteria to specific agents. The leaky vaccine approach considers that all individuals who receive a vaccine receive a proportion of protection, this style of approach lends itself well to equation-based models as it considers the population as an aggregated state.

As for the models presented here, we can take steps forward to consider whether maximising capacity and having longer interventions might not be more beneficial than small “circuit breaker” interventions when one considers the cost of hospital use and the local economy. For example, by associating a cost to hospital usage, or to an intervention in general, we can find the maximum capacity threshold to go into an intervention such that, for a specific value of $R_{0}$, we minimise the total costs by using some of the measurements we presented in this study. A similar study was conducted in [15] where they combined the COVID-19 cases from the Imperial College model [16] with typical hospital costs. They set up scenarios based around transmission, using an increase and decrease of 50% transmission at specified times to mimic interventions, and projected the costs for four and twelve weeks. Some costs of interest were the cost of capital (e.g. extra hospitals, provision of hand-washing stations), one-time costs (e.g. hiring consultants to adapt policy, prepare online training courses), the cost of commodities (e.g. extra single use masks, specific increase in drugs) and the cost of human resources (e.g. extra doctors, extra cleaners). Since the model they consider is on a national scale and uses national derived parameters, one can extend their modelling approach to regional and local levels by using our approach.

From a practical perspective, a follow up question to ask is: what about the recovery procedure? It is well known that recovering from COVID-19 is not as easy as recovering from, say, the common cold [96, 97]. Some people completely recover but invasive treatment may have caused further complications, whilst some people may continue to show effects of COVID-19 far into the future, namely suffering from long COVID [98–100]. In this case, it is natural to extend the model in this study to include, what the NHS call, Discharge to Assess, which describe the nature of the discharge of a patient and what recovery services they will need, the so-called discharge pathways. Each pathway describes the level of need of a discharged patient, each level having an associated requirement and cost. Hence the following question arises: what will the burden to healthcare across the country be in one year, five years, and so on? Understanding the pressure on discharge pathways due to COVID-19 may give an indication on recovery costs post COVID-19 infection and/or hospitalisation.

## 5 Conclusion

In this study, we have presented a computational approach for measuring the impact of healthcare demand and capacity due to surges in COVID-19 infections and hospitalisations. We have used the notion of hospital capacity as a measure for exploring intervention scenarios that will allow hospitals to predict and forecast when capacity is close to being breached and therefore allow resource allocations where necessary. The key findings are:

- We have described three different approaches to mathematically model infectious diseases, their similarities, and when each of them is appropriate to be used.
- We have demonstrated that interventions will make a significant impact on the percentage of individuals who will die as a result of COVID-19.
- We have described an easily definable and understandable method of introducing an intervention which only depends on current hospital demand and capacity.
- We have described how forecasts and interventions can be used for early warning detection systems for resource management in hospitals.
- We have shown how to calculate a threshold for optimising hospital capacity using the deterministic approach, and then demonstrated the uncertainty around this threshold. Moreover, we have shown approximately the optimal value of this threshold that results in very unlikely breaching scenarios.

With the rise of data-science over the last decade, data availability and quality has enabled data-driven modelling and research allowing for clear applications of infectious disease modelling, rather than just theoretical work. In particular, the availability of government-led data initiatives make epidemiological and public health data accessible [101]. Modelling efforts should now be conducted in such a way that allow them to be used in the future, to which we can use COVID-19 data and policy as an application and justification of the work. However, the majority of mathematical modelling publications are aimed at national level modelling with an assumption that the reader knows the standard mathematical jargon. The inherent assumptions behind the modelling decisions and the parameters that are adjusted for interventions scenarios are often not made clear. The swift wave of COVID-19 across the globe has identified the need for reliable, sensitive and validated data-driven approaches that are accessible by local authorities to make quantitative and qualitative decisions on policy. To combat this, public-policy in mind, epidemiological research groups across the UK, and in fact across the world, have been producing web-based tools to combat COVID-19 and provide ways for non-mathematicians to picture and understand the data available. A comprehensive review of different web-based tools can be found in [102]. Since these models are readily available to be used, and with the conclusion and recommendations of the Goldacre report for public healthcare management to “embrace help from other sections such as academia” [103], it is more important now more than ever that the mathematically modelling assumptions are present, visible and understandable and that the scope of the model is clear.

Our approaches are built around using a simple SEIR-D model coupled with novel statistical methods for parameter estimation and reframing the system, with the found parameters, in an agent-based approach to allow us to explore various plausible hypothetical scenarios that are of interest to the NHS local healthcare management teams and death management teams in local authorities. The theoretical and computational approach has a strong interplay between data and the model, whereby data drives the optimal parameter estimates and these in turn drive model predictions through dynamic models. Whilst this manuscript focuses on some mathematical approaches to COVID-19 and some of the questions asked by local authorities, the methodology of developing research questions is not unique to this subject. By developing and devising meaningful research questions, collaboratively between local authorities and universities, mathematical modelling should provide a bridge between institutions and organisations to answer questions of operational interest.

## Supporting information

S1 Appendix Supplementary material. Supplementary material containing the parameter estimation approach, information about data, further figures and the verification of parameters for the length of stay approach.
(PDF)
