# Supplementary material for: A hospital demand and capacity intervention approach for COVID-19
Source: PLoS One. 2023 May 3;18(5):e0283350. doi: 10.1371/journal.pone.0283350 (PMC10156009; doi:10.1371/journal.pone.0283350)
Supplement: S1 Appendix — Supplementary material containing the parameter estimation approach, information about data, further figures and the verification of parameters for the length of stay approach. (PDF) [file pone.0283350.s001.pdf]

Supplementary Material

A hospital demand and capacity intervention approach for COVID-19

James Van Yperen[1], Eduard Campillo-Funollet[2,3], Rebecca Inkpen[1], Anjum Memon[4], and Anotida Madzvamuse*[1,5,6,7]

[1]School of Mathematical and Physical Sciences, University of Sussex, Brighton, UK
[2]School of Mathematical, Statistical and Actuarial Sciences, University of Kent, Canterbury, UK
[3]Department of Mathematics and Statistics, University of Lancaster, Lancaster, UK
[4]Department of Primary Care and Public Health, Brighton and Sussex Medical School, Brighton, UK
[5]Department of Mathematics and Applied Mathematics, University of Johannesburg, Johannesburg, South Africa
[6]Department of Mathematics, University of British Columbia, Vancouver, Canada
[7]Department of Mathematics, University of Pretoria, Pretoria, South Africa

1 Data

In [1] we used specific regional datasets to calibrate the model. As part of the national COVID response, all the National Health Service (NHS) hospitals in England treating COVID-19 patients submitted a daily Situation Report (SITREP) to NHS England. The data associated to Sussex NHS Trusts was extracted and combined to weekly death counts from the Office for National Statistics (ONS) with COVID-19 reported as the underlying cause of death. To be precise, by Sussex we mean the collective term for geographies pertaining to the counties of East Sussex and West Sussex in South East England. The SITREP contained counts of daily admissions, daily discharges, and the beds occupied daily, whilst the ONS death dataset contained the number of deaths recorded outside of hospitals and the number of deaths recorded within hospitals per week. Here we regard outside of hospital as entries with "Home" as their place of death, this is due to complications with assumptions on the other categories. For example, the assumption that the individuals being modelled are well mixed is not necessarily upheld in care homes.

Whilst the ONS death data is publicly available, the SITREP data in general is not, but, given the national need for data, the UK government produced the Coronavirus Dashboard [2]. It provides users with the ability to look at different metrics of COVID-19, such as hospitalisations and deaths, for different regions and provides an API for users to download the datasets. The granularity of the data depends on the size of the region the data is required for, typically all data is available for each nation of the UK, but hospitalisations and deaths are split depending on their geographical location. Indeed, hospitalisations are recorded using NHS regions and NHS trusts, and deaths are recorded using local authority regions. The unfortunate difference between using the Coronavirus dashboard and the SITREP is that the Coronavirus dashboard does not contain the number of daily discharges, and it does not differentiate place of death like the ONS weekly death registry does. In order to apply the approach presented in [1] we adapted the Coronavirus dashboard data in the following way: we calculated the proportion of hospital COVID-19 deaths for the region being considered from the ONS weekly death registry and applied this proportion to the deaths dataset acquired from the Coronavirus Dashboard to give us a proxy dataset on deaths in hospital and deaths outside of hospital.

*Corresponding author: am823@math.ubc.ca, a.madzvamuse@sussex.ac.uk

Next, we used the deaths in hospital with the admissions and beds occupied datasets to find a proxy dataset for the discharges, since the offset of beds occupied between each day depends on the number of admissions, discharges and deaths that day. We note that this results in a very noisy discharges dataset due to noise accumulating from multiple sources. For this reason, we decided to apply a 7-day rolling average to the discharges. The Coronavirus dashboard has access to patients in mechanical ventilation beds and so we could include a compartment that describes the high-dependency unit (HDU). We decided not to do this since we do not have access to the number of patients who have died in HDU, we only have access to the number of patients who died in hospital, and so parameter identifiability would be an issue.

Other than issues of data availability, data collection is something that needs to be considered when calibrating mathematical models. In particular, one needs to consider whether underreporting is a significant factor from the data and, if so, how does on take that into consideration in the calibration process. In this manuscript, we are assuming that underreporting is not significant in order to not overly complicate the objective of the study, however we acknowledge this is not necessarily true. In particular, one should take into account the following

- a patient was admitted to hospital due to something unrelated to COVID-19, but also tested positive for COVID-19 on admission;
- a patient died in hospital due to something unrelated to COVID-19, but also had COVID-19 when they died;
- a patient was infected whilst in hospital (nosocial infections);
- a patient died in the community due to something unrelated to COVID-19, but also had COVID-19 when they died.

Underreporting was the main reason why we did not use cases data when conducting our study in [1], and thus here as well. Whilst the appearance of an underreporting parameter in the data is not hard to reason, the derivation of the observational model and understanding what parameters are identifiable is not trivial. We have studied this when considering a simple SIR model here [3]. In other modelling approaches for COVID-19, groups estimate underreporting parameters using periods of data that have a large number of tests with a small positivity rate and then this value to forecast and backcast, or use the case-fatality ratio to estimate cases, see [4, 5]. For an extensive review of ways to deal with underreporting for infectious disease modelling, see [6] and references therein.

In the codebase for the manuscript, we have given details on where we found the data and how to use the Coronavirus Dashboard Application Programming Interface (API). Namely, in the folder named "parameter_estimation\data_management", we have written a markdown file called "data_sources.md" which details the data downloaded from the Coronavirus Dashboard and the ONS. For the Coronavirus Dashboard API, we have written a Python script called "coronavirus_dashboard_data.py" which demonstrates how to use the Python requests library and Pandas library to obtain the data and store it [7, 8]. In order to obtain the relevant information to use the API, such as metrics and location codes, readers need to look in the script "run_parameter_estimation.py", which can be found in the root of the codebase.

2 Parameter estimation

We now briefly explain the calibration procedure presented in [1]. For ease of exposition, we restate the SEIR-D model Eq (1) to Eq (9) in the main manuscript here

$$\dot{S} = -\beta \frac{U+I}{N} S, \qquad t \in (0,T], \qquad S(0) = S_0, \tag{1}$$
$$\dot{E} = \beta \frac{U+I}{N} S - \gamma_E E, \qquad t \in (0,T], \qquad E(0) = E_0, \tag{2}$$
$$\dot{U} = p\,\gamma_E E - \gamma_U U, \qquad t \in (0,T], \qquad U(0) = U_0, \tag{3}$$
$$\dot{I} = (1-p)\gamma_E E - \gamma_I I, \qquad t \in (0,T], \qquad I(0) = I_0, \tag{4}$$
$$\dot{H} = \gamma_I I - (\gamma_H + \mu_H)H, \qquad t \in (0,T], \qquad H(0) = H_0 \tag{5}$$
$$\dot{R}_U = (1 - m_U)\gamma_U U, \qquad t \in (0,T], \qquad R_U(0) = R_{U,0}, \tag{6}$$
$$\dot{R}_H = \gamma_H H, \qquad t \in (0,T], \qquad R_H(0) = R_{H,0}, \tag{7}$$
$$\dot{D}_U = m_U \gamma_U U, \qquad t \in (0,T], \qquad D_U(0) = D_{U,0}, \tag{8}$$
$$\dot{D}_H = \mu_H H, \qquad t \in (0,T], \qquad D_H(0) = D_{H,0}. \tag{9}$$

First, we utilise the linear relationship between the model description of hospital discharges and hospital deaths and use linear regression analysis to calculate the ratio of discharges to deaths. One can see that, in terms of the model and its parameters, the daily discharges can be written as

$$Dis(t) := \gamma_H \int_{t-1}^{t} H(s)\,\mathrm{d}s, \tag{10}$$

and the daily hospital deaths can be written as

$$Dth_H(t) := \mu_H \int_{t-1}^{t} H(s)\,\mathrm{d}s, \tag{11}$$

which means we can estimate $\gamma_H \mu_H^{-1}$. Next, we rewrite equations Eq (1) to Eq (5) in terms of the data, we call this the "observational" model, whereby Eq (6) to Eq (9) are not considered since they are cumulative representations of the compartments in Eq (1) to Eq (5). One can see that the daily admissions can be written as

$$Adm(t) := \gamma_I \int_{t-1}^{t} I(s)\,\mathrm{d}s, \tag{12}$$

and the daily deaths outside of hospital is

$$Dth_U(t) := \gamma_U m_U \int_{t-1}^{t} U(s)\,\mathrm{d}s. \tag{13}$$

Thus, the observational model, as presented in [1], is

$$\dot{H} = \gamma_I I - \gamma_H \left(1 + \frac{\mu_H}{\gamma_H}\right) H, \tag{14}$$
$$\dot{U} = \frac{p}{1-p}\left(\dot{I} + \gamma_I I\right) - \gamma_U U, \tag{15}$$
$$\dddot{I} = \left(\ddot{I} + (\gamma_E + \gamma_I)\dot{I} + \gamma_E \gamma_I I\right)\left[\frac{p\gamma_I I}{1-p} + \frac{\dot{I}}{1-p} - \gamma_U U - \frac{\beta}{N}(U+I)^2\right](U+I)^{-1}$$
$$- (\gamma_E + \gamma_I)\ddot{I} - \gamma_E \gamma_I \dot{I}. \tag{16}$$

We solve the observational model, compute Eq (10), Eq (12) and Eq (13) and compare against the datasets given. We use a maximum likelihood estimation approach by minimising a negative log-likelihood with some constraints on the initial conditions and on the effective reproduction number.

These constraints are needed to keep the feasible region of the parameters close to the realistic sets. Without these constraints, the peak of the datasets could be explained by being close to herd immunity (i.e., a lot of infections have already occurred before the lockdown) or a large value of $\mathcal{R}_t$ combined with a small value of p. We note here that Eq (11) does not need to be used in the observational model due to the linear regression and the fact that the resulting log-likelihood functions from the regression and observational model are independent. This means that there is one less parameter to infer from solving the observational model.

3 Results using interventions

3.1 Branching model results

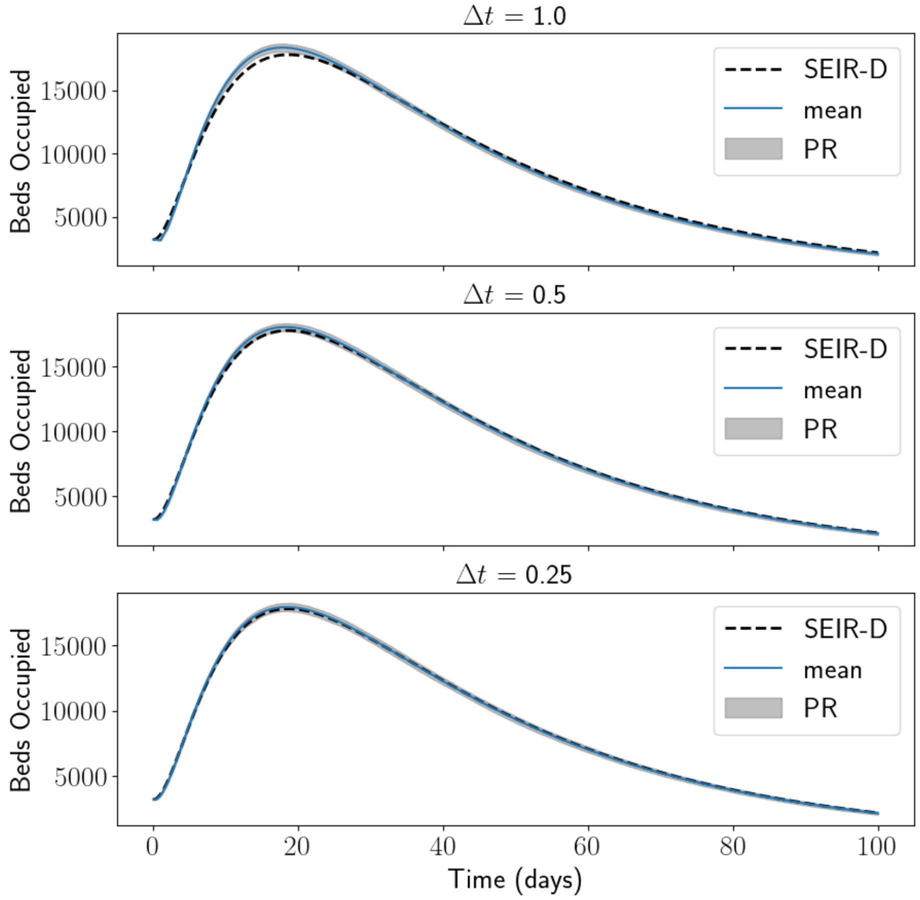

Figure 1: Beds occupied using the branching process, using the England parameters and initial conditions with varied values of Δt. The mean of the branching process matches the SEIR-D model results well for all values of Δt. PR stands for percentile range.

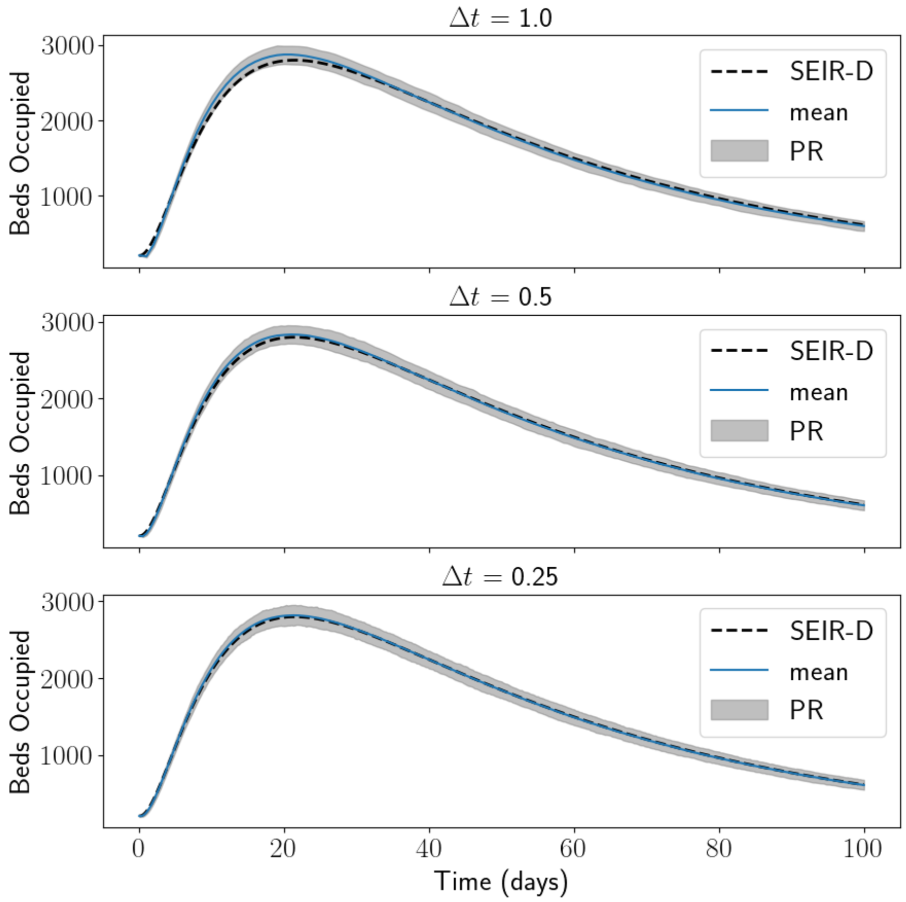

Figure 2: Beds occupied using the branching process, using the North West parameters and initial conditions with varied values of Δt. The mean of the branching process matches the SEIR-D model results well for all values of Δt. PR stands for percentile range.

3.2 LoS model results

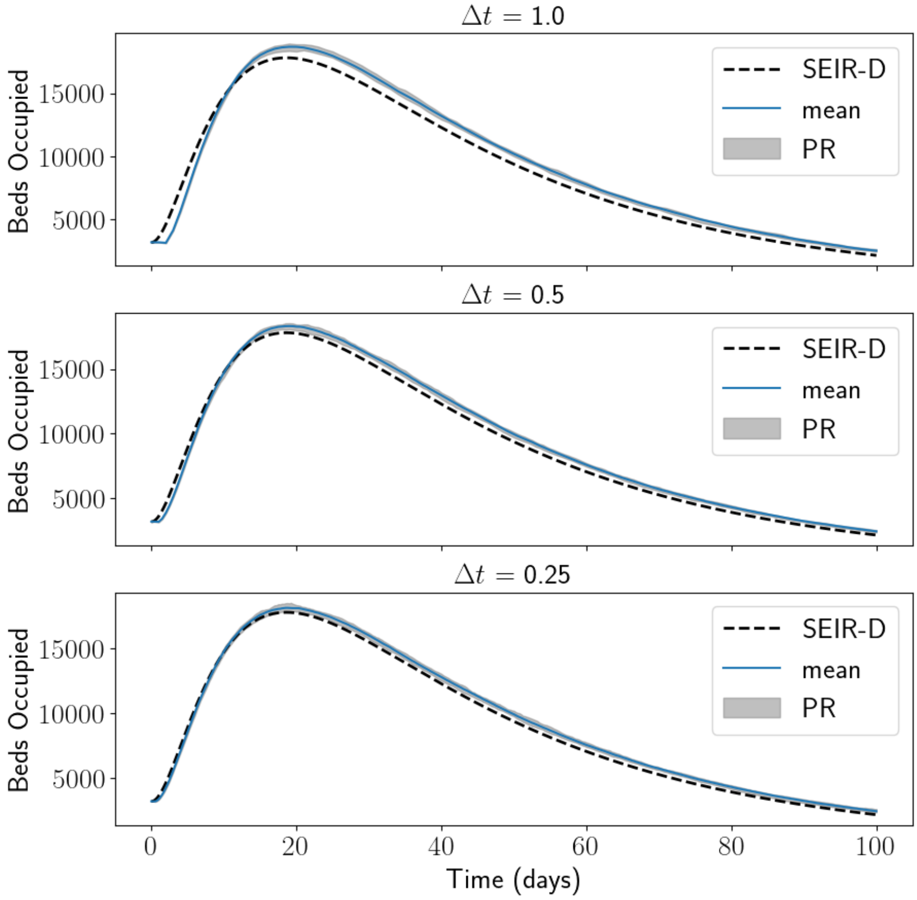

Figure 3: Beds occupied using the length of stay approach, using England parameters and initial conditions with varied values for Δt. The mean of the branching process matches the SEIR-D results well for all values of Δt.

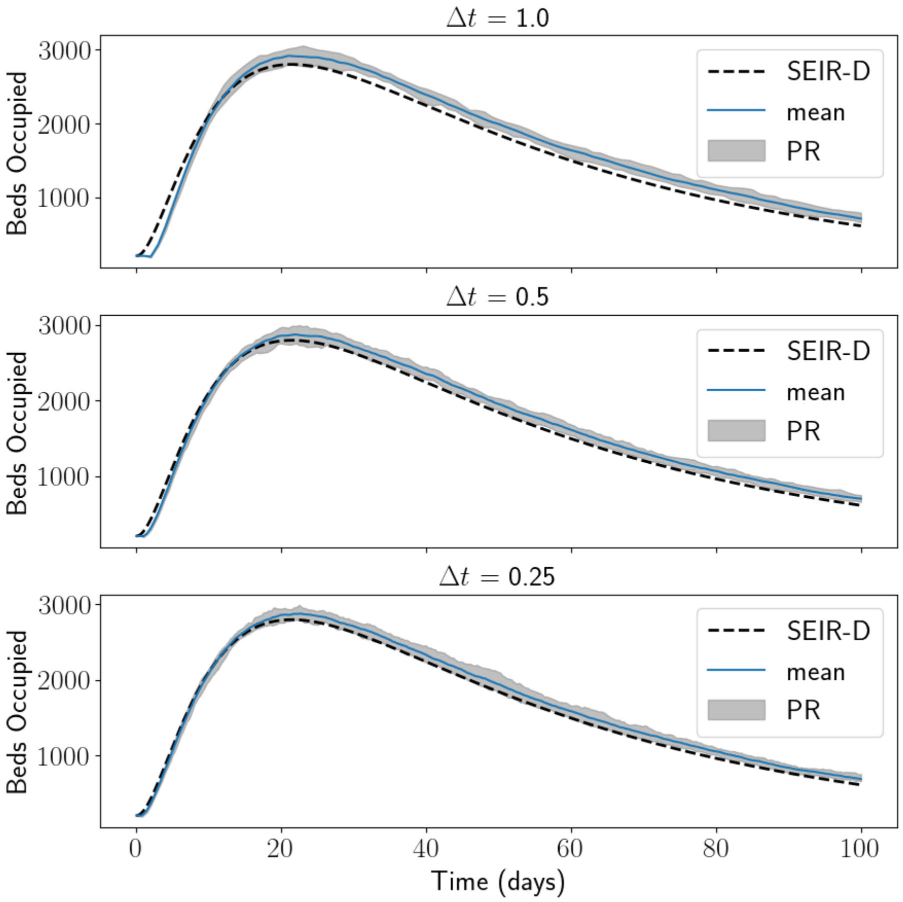

Figure 4: Beds occupied using the length of stay approach, using North West parameters and initial conditions with varied values for Δt. The mean of the branching process matches the SEIR-D results well for all values of Δt.

3.3 The do-nothing approach results

Table 1: The do-nothing approach comparing the SEIR-D model with the branching process using the England parameters. Displaying the maximum number of beds occupied (as a percentage of the population) and what day the simulation reaches that maximum, taking $\Delta t = 0.25$. BM stands for the mean of the results from branching model and PR is the percentile range.

	**Max beds occupied** (%N)			**Peak of beds occupied (day)**		
$\mathcal{R}_0$	SEIR-D	BM	(PR)	SEIR-D	BM	(PR)
1.3	0.145%	0.145%	(0.144%, 0.147%)	168.3	168.8	(164.0, 172.8)
1.4	0.222%	0.223%	(0.221%, 0.224%)	143.6	144.1	(141.3, 147.0)
1.5	0.304%	0.304%	(0.303%, 0.306%)	126.3	126.8	(125.0, 128.8)
1.6	0.386%	0.387%	(0.385%, 0.389%)	113.6	114.2	(112.5, 115.8)
1.7	0.466%	0.468%	(0.466%, 0.470%)	103.7	104.3	(103.3, 105.8)
1.8	0.544%	0.546%	(0.544%, 0.548%)	95.6	96.5	(95.5, 97.5)
1.9	0.619%	0.621%	(0.619%, 0.624%)	89.5	90.1	(89.3, 91.0)
2.0	0.690%	0.692%	(0.690%, 0.695%)	84.1	84.7	(84.0, 85.5)

Table 2: The do-nothing approach comparing the SEIR-D model with the branching process using the North West parameters. Displaying the maximum number of beds occupied (as a percentage of the population) and what day the simulation reaches that maximum, taking $\Delta t = 0.25$. BM stands for the mean of the results from branching model and PR is the percentile range.

	**Max beds occupied** (%N)			**Peak of beds occupied (day)**		
$\mathcal{R}_0$	SEIR-D	BM	(PR)	SEIR-D	BM	(PR)
1.3	0.140%	0.141%	(0.138%, 0.144%)	168.3	168.8	(160.7, 176.8)
1.4	0.214%	0.216%	(0.212%, 0.220%)	143.6	144.1	(138.5, 149.8)
1.5	0.293%	0.295%	(0.290%, 0.300%)	126.4	126.9	(122.8, 131.0)
1.6	0.372%	0.374%	(0.369%, 0.380%)	113.6	114.2	(110.0, 117.5)
1.7	0.450%	0.452%	(0.446%, 0.459%)	103.8	104.3	(101.8, 106.8)
1.8	0.525%	0.528%	(0.522%, 0.535%)	95.9	96.5	(94.5, 98.8)
1.9	0.598%	0.601%	(0.594%, 0.609%)	89.5	90.1	(88.0, 92.0)
2.0	0.666%	0.670%	(0.662%, 0.677%)	84.1	84.7	(83.0, 86.5)

3.4 Fixing the limit on demand results

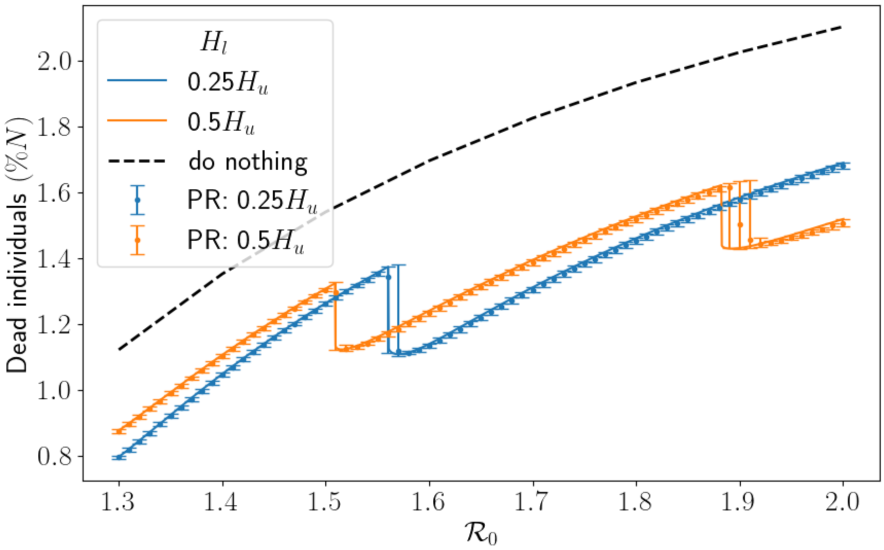

Figure 5: Percentage of dead individuals corresponding to an $\mathcal{R}_0$ value using the hospital capacity intervention approach and the England parameters. Here we have fixed $H_l := 0.25H_u$ or $H_l := 0.5H_u$, and set $\Delta t = 0.5$. The thick line represents the results from the SEIR-D model, and the error bars depict the result from the branching model. The black dashed line depicts the associated percentage of dead individuals using the do-nothing approach. PR is the percentile range from the branching model.

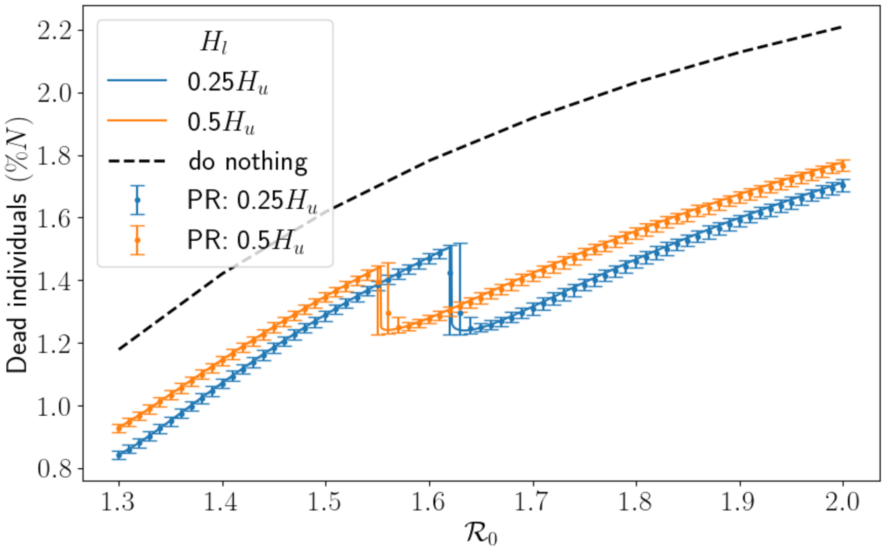

Figure 6: Percentage of dead individuals corresponding to an $\mathcal{R}_0$ value using the hospital capacity intervention approach and the North West parameters. Here we have fixed $H_l := 0.25H_u$ or $H_l := 0.5H_u$, and set $\Delta t = 0.5$. The thick line represents the results from the SEIR-D model, and the error bars depict the result from the branching model. The black dashed line depicts the associated percentage of dead individuals using the do-nothing approach. PR is the percentile range from the branching model.

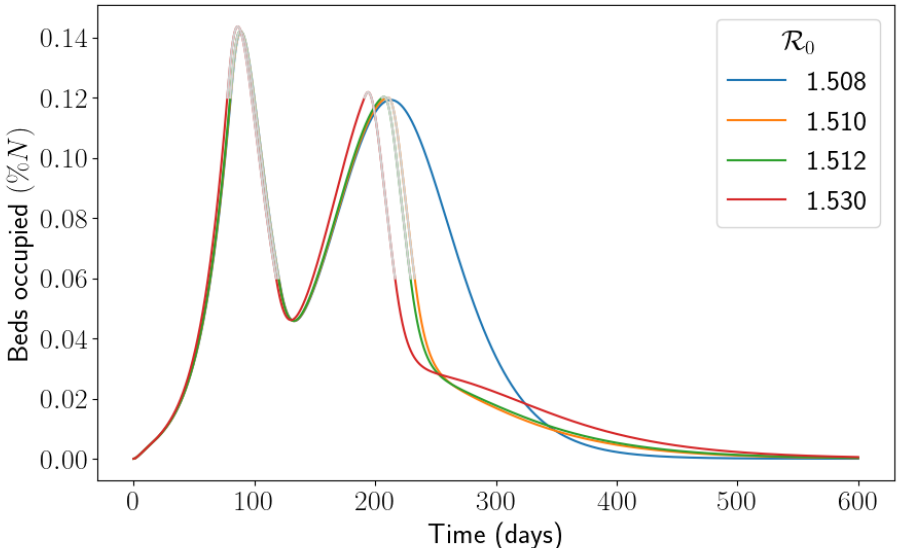

Figure 7: Percentage of patients in hospitals per day using the hospital capacity intervention approach and the England parameters. Here we have fixed $H_l := 0.5H_u$. The grey lines represent the times when the simulation is in an intervention. We note that we have truncated the simulation to make visualisation easier.

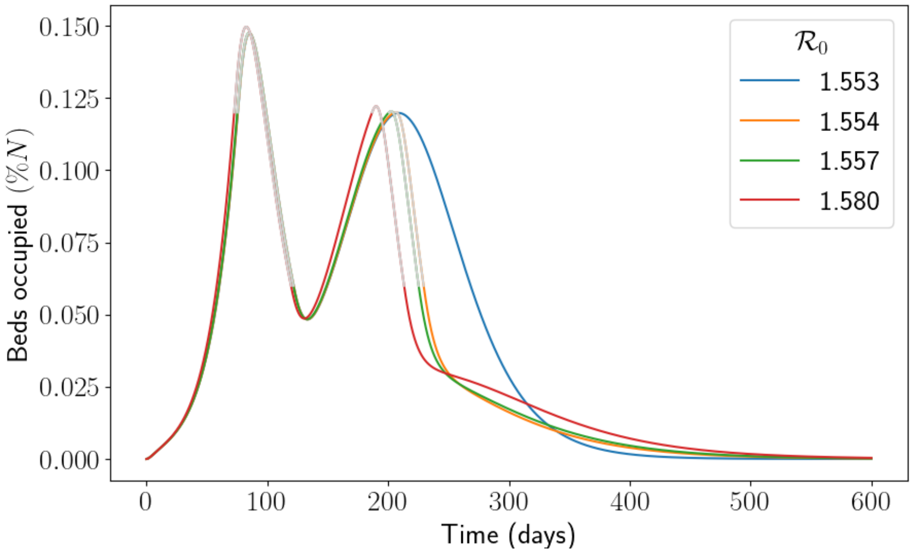

Figure 8: Percentage of patients in hospitals per day using the hospital capacity intervention approach and the North West parameters. Here we have fixed $H_l := 0.5H_u$. The grey lines represent the times when the simulation is in an intervention. We note that we have truncated the simulation to make visualisation easier.

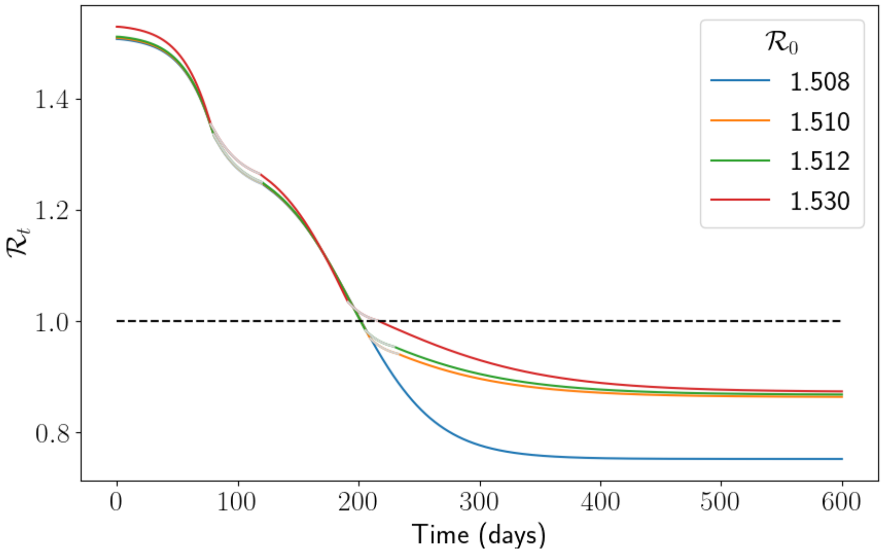

Figure 9: $\mathcal{R}_t$ per day using the hospital capacity intervention approach and the South East parameters. Here we have fixed $H_l := 0.5H_u$. The grey lines represent the times when the simulation is in an intervention. The black dashed line represents herd immunity $\mathcal{R}_t = 1$. We note that we have truncated the simulation to make visualisation easier.

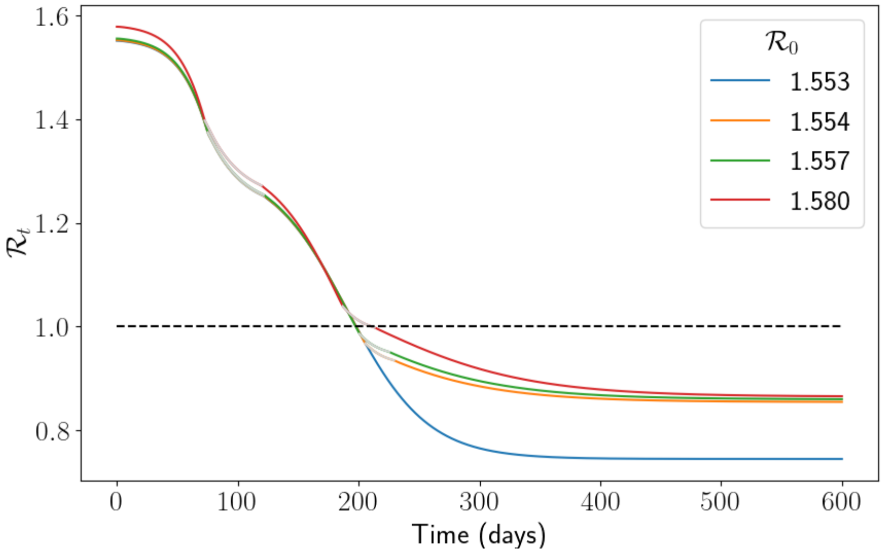

Figure 10: $\mathcal{R}_t$ per day using the hospital capacity intervention approach and the South East parameters. Here we have fixed $H_l := 0.5H_u$. The grey lines represent the times when the simulation is in an intervention. The black dashed line represents herd immunity $\mathcal{R}_t = 1$. We note that we have truncated the simulation to make visualisation easier.

3.5 Varying lower limit of demand results

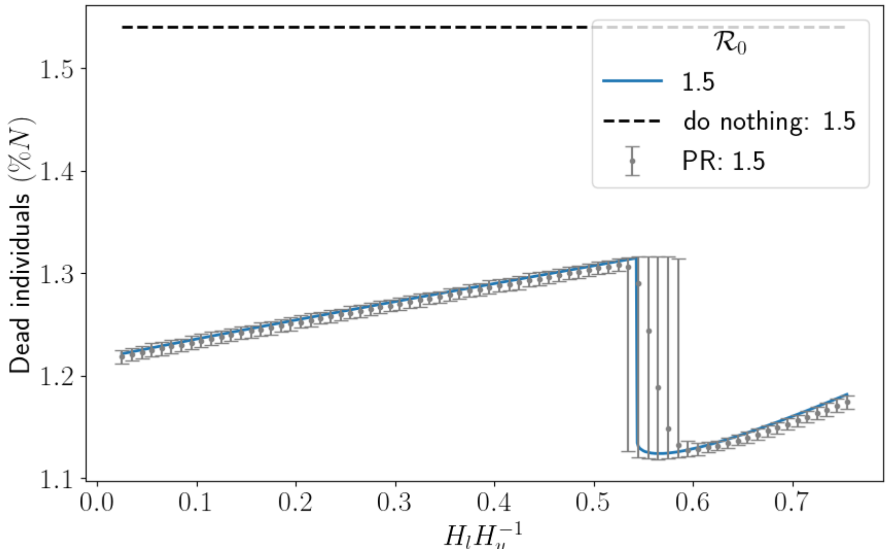

Figure 11: Percentage of dead individuals corresponding to an $\mathcal{R}_0$ value using the hospital capacity intervention approach and the England parameters. Here we have fixed $\mathcal{R}_0 := 1.5$ and set $\Delta t = 0.5$. The thick line represents the results from the SEIR-D model, and the error bars depict the result from the branching model. The black dashed line depicts the associated percentage of dead individuals using the do-nothing approach. PR is the percentile range from the branching model.

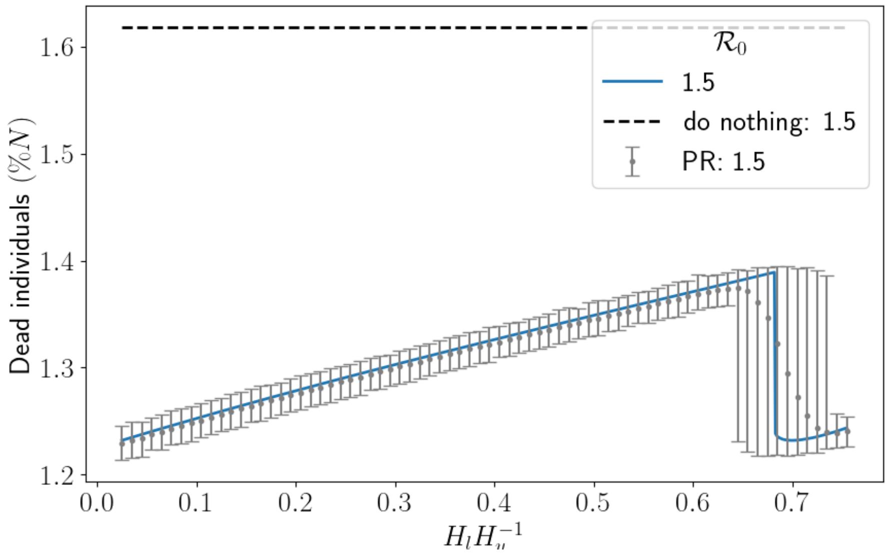

Figure 12: Percentage of dead individuals corresponding to an $\mathcal{R}_0$ value using the hospital capacity intervention approach and the North West parameters. Here we have fixed $\mathcal{R}_0 := 1.5$ and set $\Delta t = 0.5$. The thick line represents the results from the SEIR-D model, and the error bars depict the result from the branching model. The black dashed line depicts the associated percentage of dead individuals using the do-nothing approach. PR is the percentile range from the branching model.

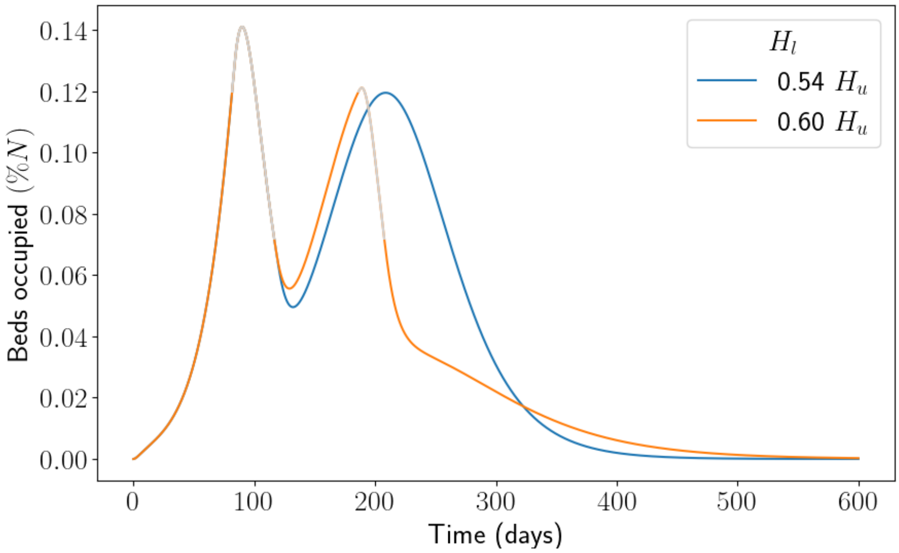

Figure 13: Percentage of patients in hospitals per day using the hospital capacity intervention approach and the England parameters. Here we have fixed $\mathcal{R}_0 = 1.5$. The grey lines represent the times when the simulation is in an intervention. We note that we have truncated the simulation to make visualisation easier.

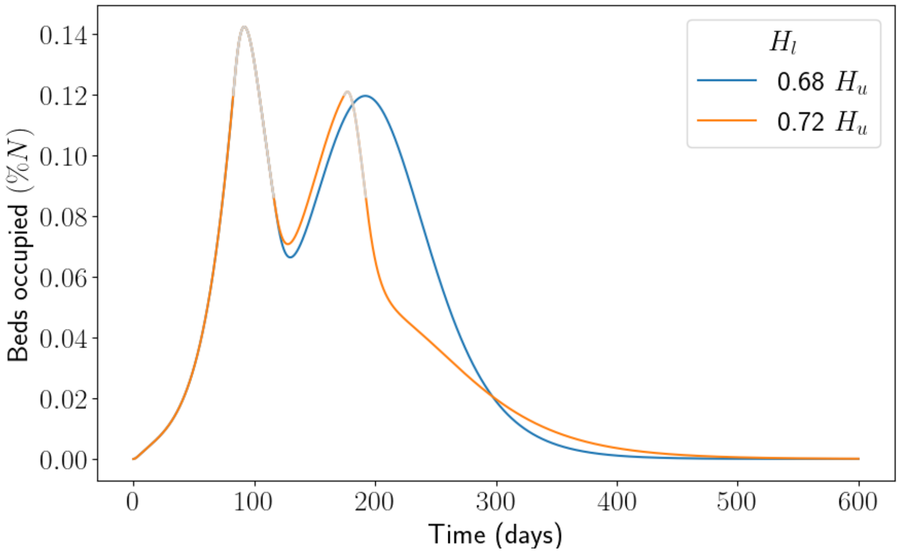

Figure 14: Percentage of patients in hospitals per day using the hospital capacity intervention approach and the North West parameters. Here we have fixed $\mathcal{R}_0 = 1.5$. The grey lines represent the times when the simulation is in an intervention. We note that we have truncated the simulation to make visualisation easier.

3.6 Uncertainty quantification of breaches

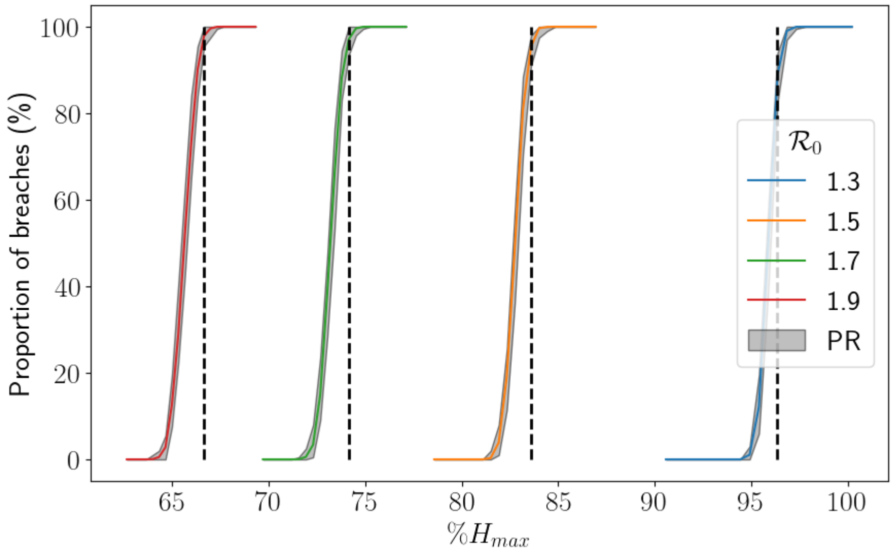

Figure 15: Proportion of breaches against different proportions of H_u associated to the value of $\mathcal{R}_0$, which have then been converted into a proportion of H_{max}, using the hospital capacity intervention approach (finishing after one intervention) and the England parameters, with $\Delta t = 0.25$. PR stands for the percentile range from the branching model and the thick lines represent the mean from the branching model. The black dashed line represents the value of H_u associated to $\mathcal{R}_0$ found in Fig 18 in the manuscript.

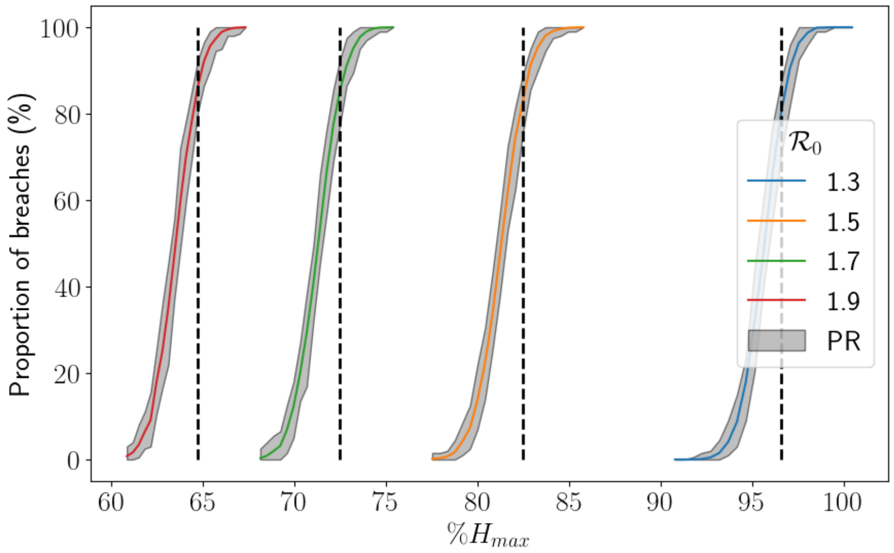

Figure 16: Proportion of breaches against different proportions of H_u associated to the value of $\mathcal{R}_0$, which have then been converted into a proportion of H_{max}, using the hospital capacity intervention approach (finishing after one intervention) and the South East parameters, with $\Delta t = 0.25$. PR stands for the percentile range from the branching model and the thick lines represent the mean from the branching model. The black dashed line represents the value of H_u associated to $\mathcal{R}_0$ found in Fig 18 in the manuscript.

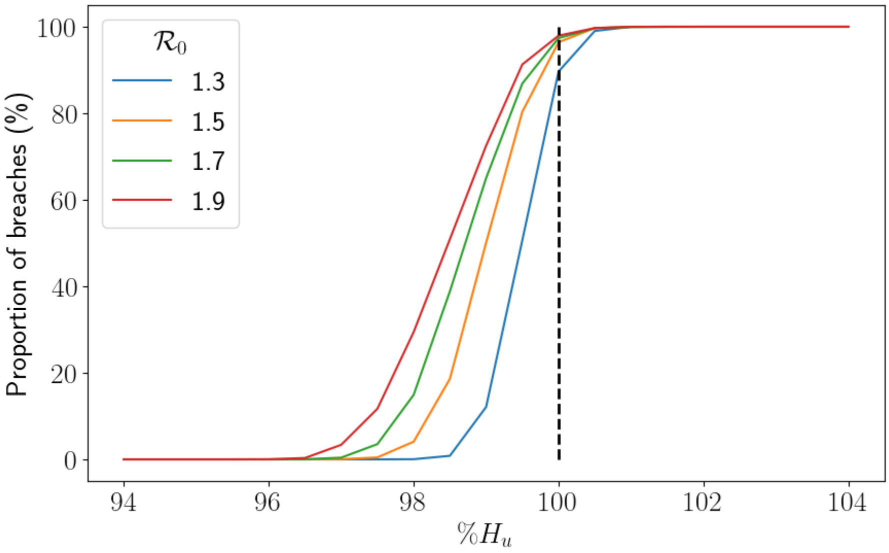

Figure 17: Proportion of breaches against different proportions of H_u associated to the value of $\mathcal{R}_0$ using the hospital capacity intervention approach (finishing after one intervention) and the England parameters, with $\Delta t = 0.25$. PR stands for the percentile range from the branching model and the thick lines represent the mean from the branching model.

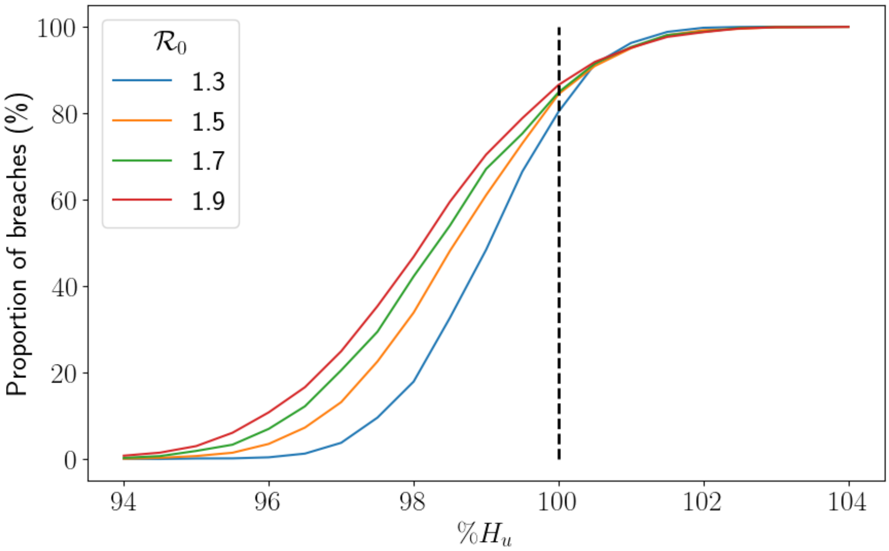

Figure 18: Proportion of breaches against different proportions of H_u associated to the value of $\mathcal{R}_0$ using the hospital capacity intervention approach (finishing after one intervention) and the North West parameters, with $\Delta t = 0.25$. PR stands for the percentile range from the branching model and the thick lines represent the mean from the branching model.

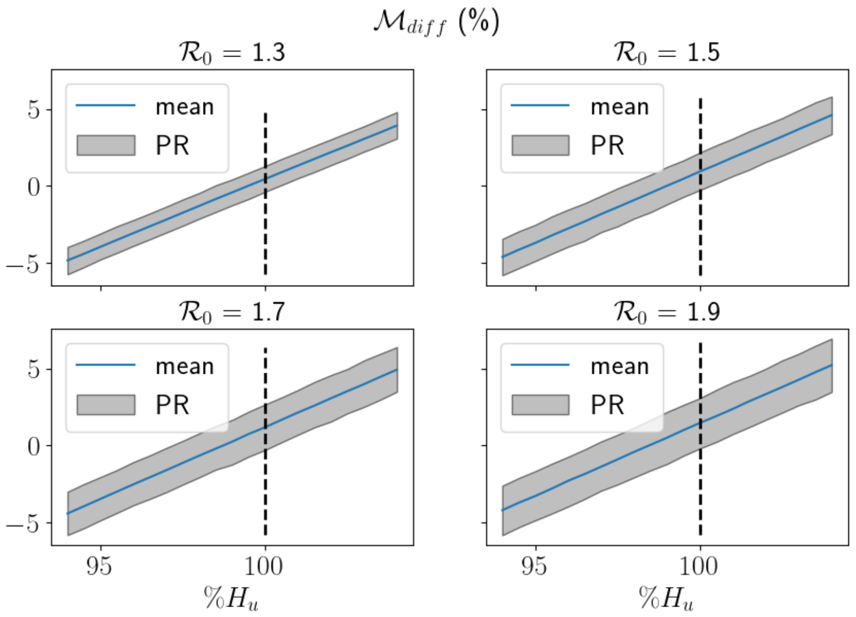

Figure 19: $\mathcal{M}_{diff}$ against different proportions of H_u associated to the value of $\mathcal{R}_0$ using the hospital capacity intervention approach (finishing after one intervention) and the England parameters, with $\Delta t = 0.25$. PR stands for the percentile range from the branching model and the thick lines represent the mean from the branching model.

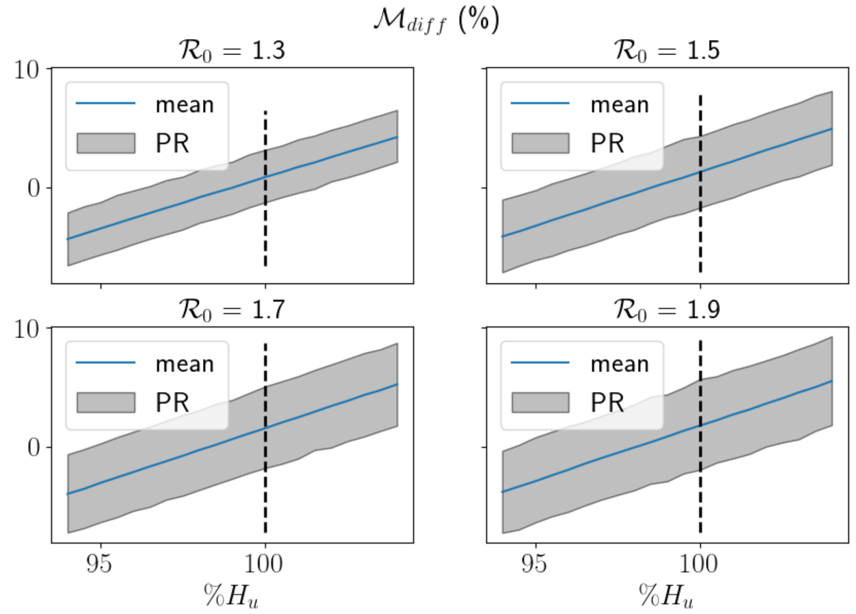

Figure 20: $\mathcal{M}_{diff}$ against different proportions of H_u associated to the value of $\mathcal{R}_0$ using the hospital capacity intervention approach (finishing after one intervention) and the North West parameters, with $\Delta t = 0.25$. PR stands for the percentile range from the branching model and the thick lines represent the mean from the branching model.

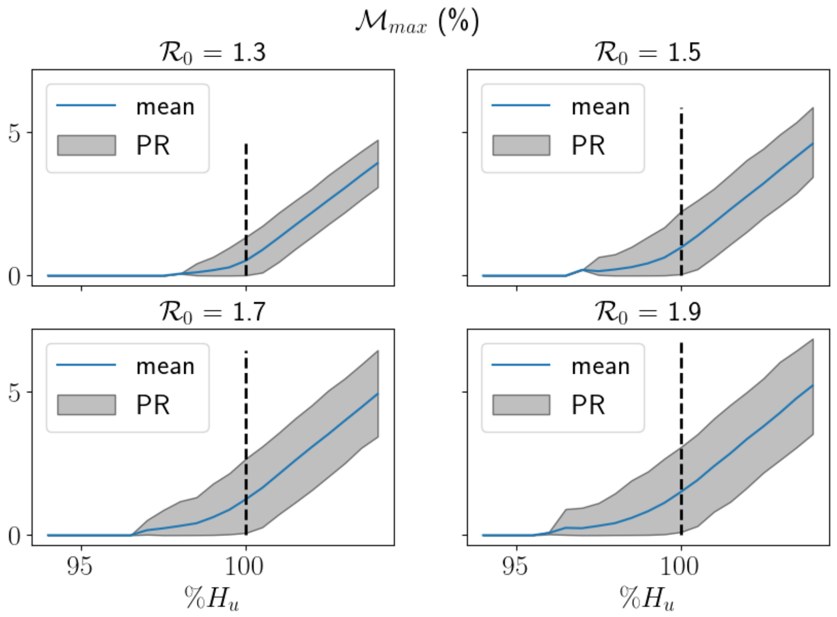

Figure 21: $\mathcal{M}_{max}$ against different proportions of H_u associated to the value of $\mathcal{R}_0$ using the hospital capacity intervention approach (finishing after one intervention) and the England parameters, with $\Delta t = 0.25$. PR stands for the percentile range from the branching model and the thick lines represent the mean from the branching model. The black dashed line represents the value of H_u associated to $\mathcal{R}_0$.

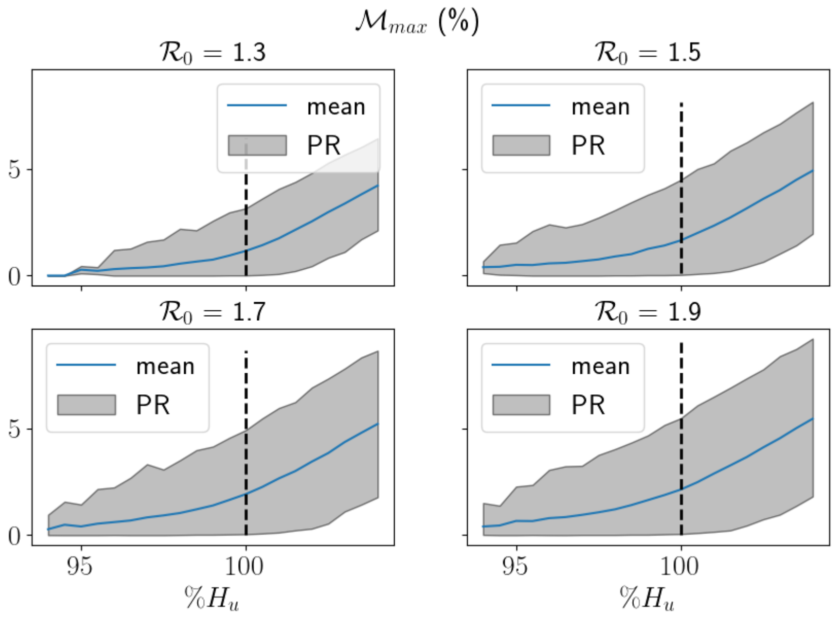

Figure 22: $\mathcal{M}_{max}$ against different proportions of H_u associated to the value of $\mathcal{R}_0$ using the hospital capacity intervention approach (finishing after one intervention) and the North West parameters, with $\Delta t = 0.25$. PR stands for the percentile range from the branching model and the thick lines represent the mean from the branching model. The black dashed line represents the value of H_u associated to $\mathcal{R}_0$.

4 Verification of parameters for the agent-based approach

In order to verify that the length of stay agent-based approach was producing the same output on average as the SEIR-D model (and thus the data) without having to do any parameter estimation, we chose to check the following criteria:

- is the mean of the output of the length of stay approach almost indistinguishable to the output of the SEIR-D model?
- is the mean of the time spent in the states E, U and I close to the inverse of the associated rate parameter?
- is the proportion of people going into U, D_U and D_H close to the associated probability parameters?

The first criterion was measured by plotting both approaches, the second criterion was calculated by measuring the frequency of the length of stay of each agent in each compartment during the simulation, and the third criterion was calculated by counting the number of agents who went along each decision.

When conducting this investigation we noticed that the lenght of stay approach quite significantly overestimated the result, as can be seen in Fig 23, but converges towards the SEIR-D approach as we reduce Δt. When checking the rate parameters, we noticed that the observed mean was approximately $0.5\Delta t$ greater than the expected mean from the fitted parameters, which can be seen in Fig 24 and Table 3 both by value and by the fact that the red line (the expected probability density function) mostly goes through the next histogram column at the corner. We also noticed that this was independent of the number of Monte Carlo realisations. This led us to add on a correction term to the fitted parameters to reduce the observed mean towards the true value, namely by solving

$$\frac{1}{\gamma + c} = \frac{1}{\gamma} - \frac{\Delta t}{2},$$

which rearranges to Eq (26) in the manuscript. We note that $c(\gamma; \Delta t) \to 0$ as $\Delta t \to 0$. Using this correction term, we see that the output of the length of stay approach matches the SEIR-D approach better in Fig 25 and the observed means for the parameters are significantly more accurate in Fig 26 and Table 4. It is important to stress that whilst we do see convergence as Δt tends to 0, significantly more computational power is needed when reducing Δt. By adding the correction term, we can use a larger time step, and thus less computational power, whilst still maintaining accurate results. This is particularly important when one could consider using a agent-based approach to model a complex phenomena, say one which does not have an obvious equation-based approach, and still be able to conduct parameter estimation. In Tables 3 and 4 we also demonstrate a 95% confidence interval around the estimated parameters.

Table 3: Observed values, with confidence intervals, of the rate parameters using different values of Δt to compare the observed value against the true value given the state and fitted parameter without the correction term.

	Δt (day)			
Parameter	1	0.5	0.25	True value
γ_E^{-1}	4.954<br>(4.945, 4.963)	4.683<br>(4.675, 4.692)	4.554<br>(4.546, 4.562)	4.428
γ_U^{-1}	5.204<br>(5.078, 5.330)	4.907<br>(4.795, 5.020)	4.833<br>(4.726, 4.941)	4.662

As for the probability parameters, we noticed that as the number of Monte Carlo realisations increased, the closer the probabilities got to the SEIR-D parameter, which can be seen in Table 5. Intuitively, this is to be expected as the more decisions being made, the closer the probability should be approximated. One also notices that the probabilities can be calculated at the end of the simulation by manipulating the SEIR-D approach in the following way. To obtain m_U, one integrates (6) and rearranges to find

$$\gamma_U \int_0^T U(s)\,\mathrm{d}s = \frac{1}{1 - m_U}(R_U(T) - R_U(0)),$$

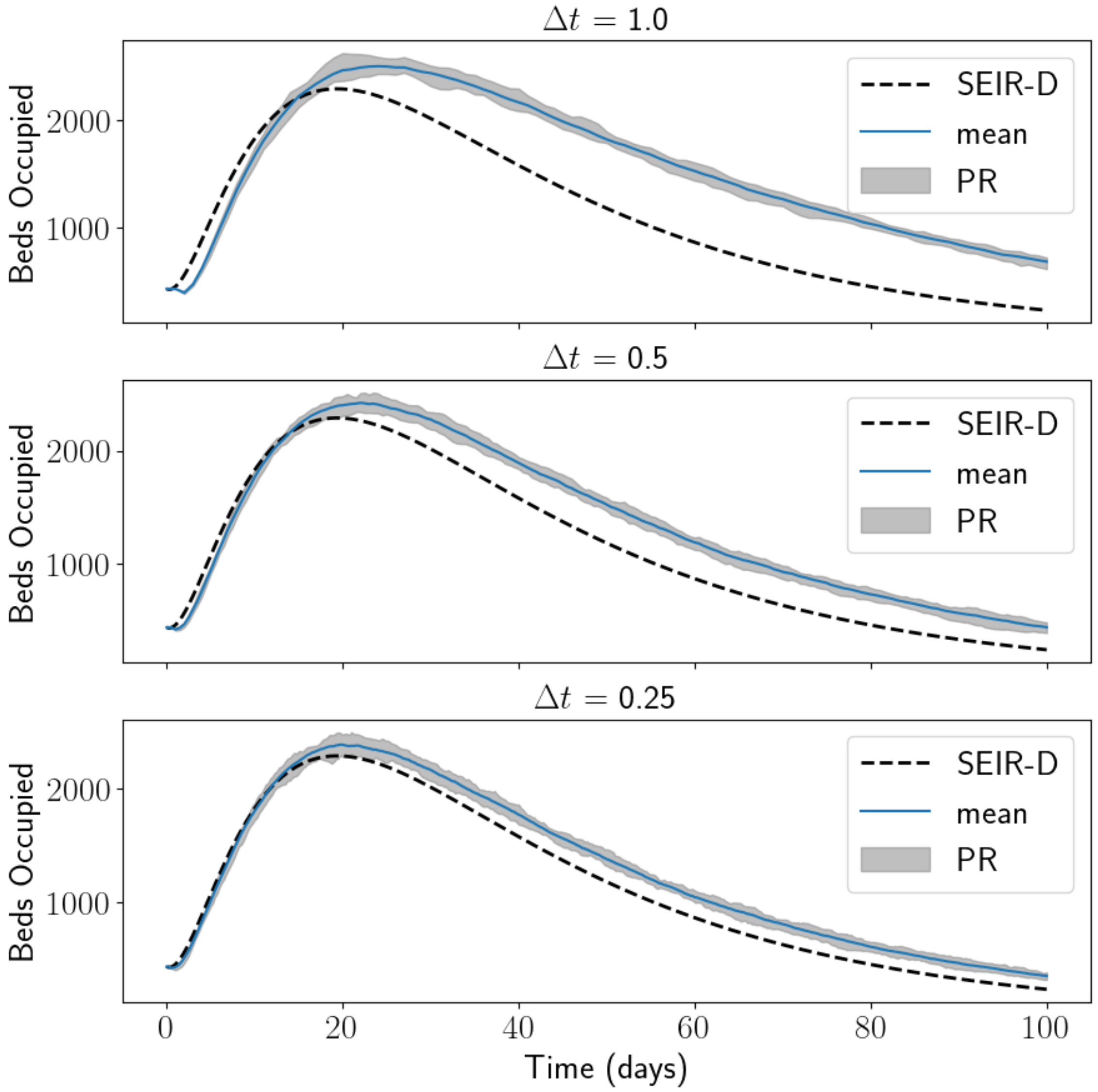

Figure 23: Beds occupied using the length of stay approach without the correct term, using South East parameters and initial conditions with varied values of Δt. The mean of the length of stay approach is starting to get close to the SEIR-D result for small Δt. PR stands for the percentile range.

Table 4: Observed values, with confidence intervals, of the rate parameters using different values of Δt to compare the observed value against the true value given the state and fitted parameter with the correction term.

	Δt (day)			
Parameter	1	0.5	0.25	True value
γ_E^{-1}	4.451<br>(4.442, 4.460)	4.435<br>(4.427, 4.444)	4.434<br>(4.426, 4.443)	4.428
γ_U^{-1}	4.656<br>(4.542, 4.770)	4.680<br>(4.573, 4.788)	4.770<br>(4.664, 4.877)	4.662

which, by integrating (8), inserting above and rearranging, gives

$$m_U = \frac{D_U(T) - D_U(0)}{R_U(T) - R_U(0) + D_U(T) - D_U(0)}.$$

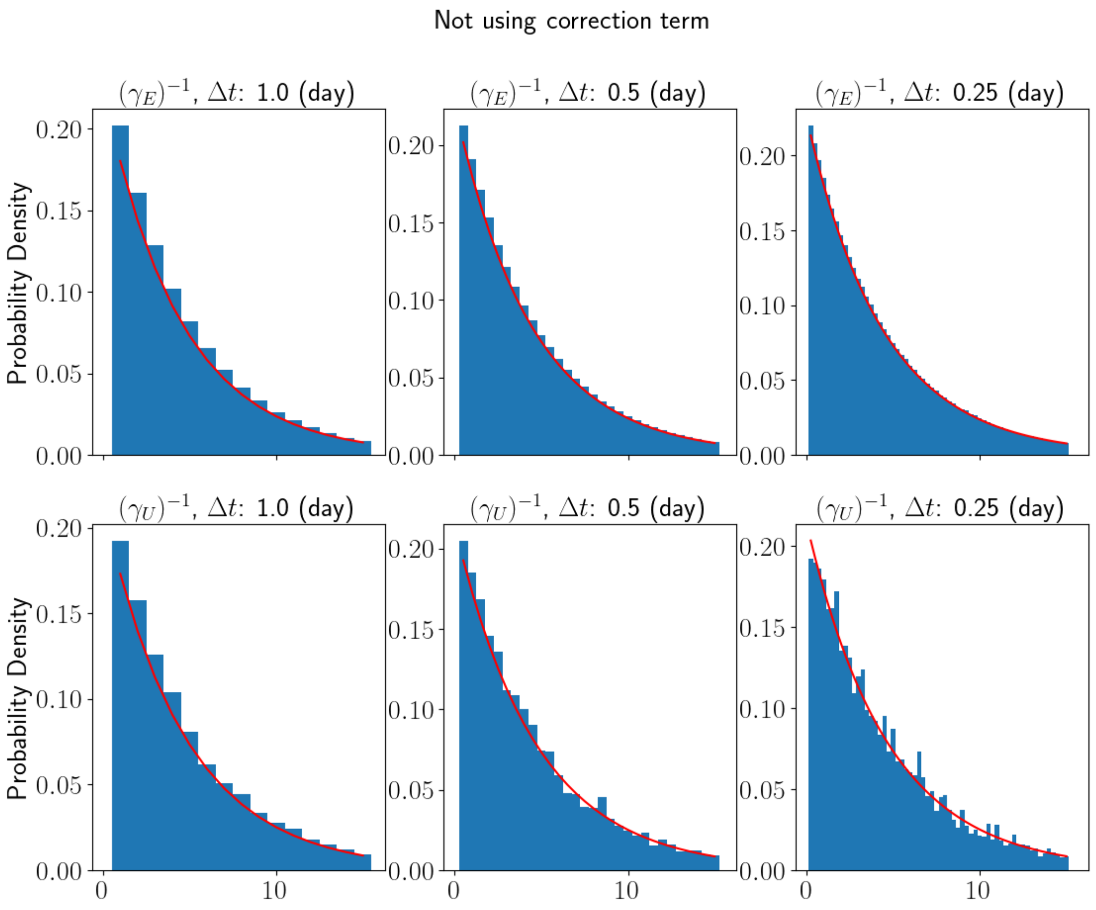

Figure 24: Frequency density of lengths of stay of agents in states E and U without the use of the correction term. The titles of each plot depict the associated parameter of the state in consideration and the time step (in days). The first row corresponds to lengths of time agents spend in the E state, which should correspond to an exponential distribution with mean γ_E, and the second row corresponds to lengths of time agents spend in the U state, which should correspond to an exponential distribution with mean γ_U. The first column uses a time unit of 1 day, the second column uses a time unit half a day and the last column uses a time unit of a quarter of a day.

One can apply the same idea to get

$$m_H = \frac{D_H(T) - D_H(0)}{R_H(T) - R_H(0) + D_H(T) - D_H(0)},$$

and

$$p = \frac{U(T) - U(0) + \gamma_U \int_0^T U(s)\,\mathrm{d}s}{U(T) - U(0) + \gamma_U \int_0^T U(s)\,\mathrm{d}s + I(T) - I(0) + \gamma_I \int_0^T I(s)\,\mathrm{d}s}.$$

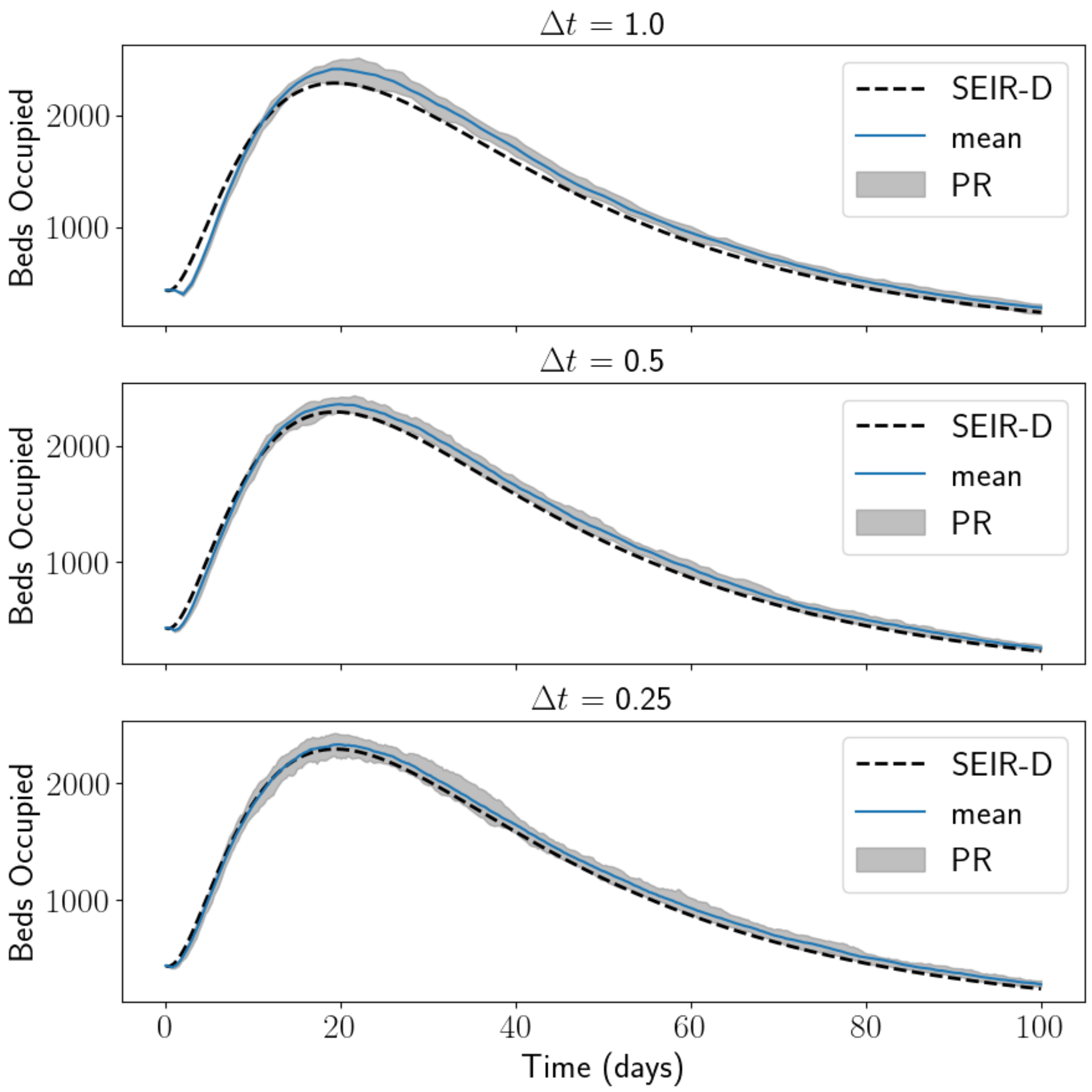

Figure 25: Beds occupied using the length of stay approach, using the South East parameters and initial conditions with varied values for Δt. The mean of the length of stay approach matches the SEIR-D results well for all values of Δt. PR stands for the percentile range.

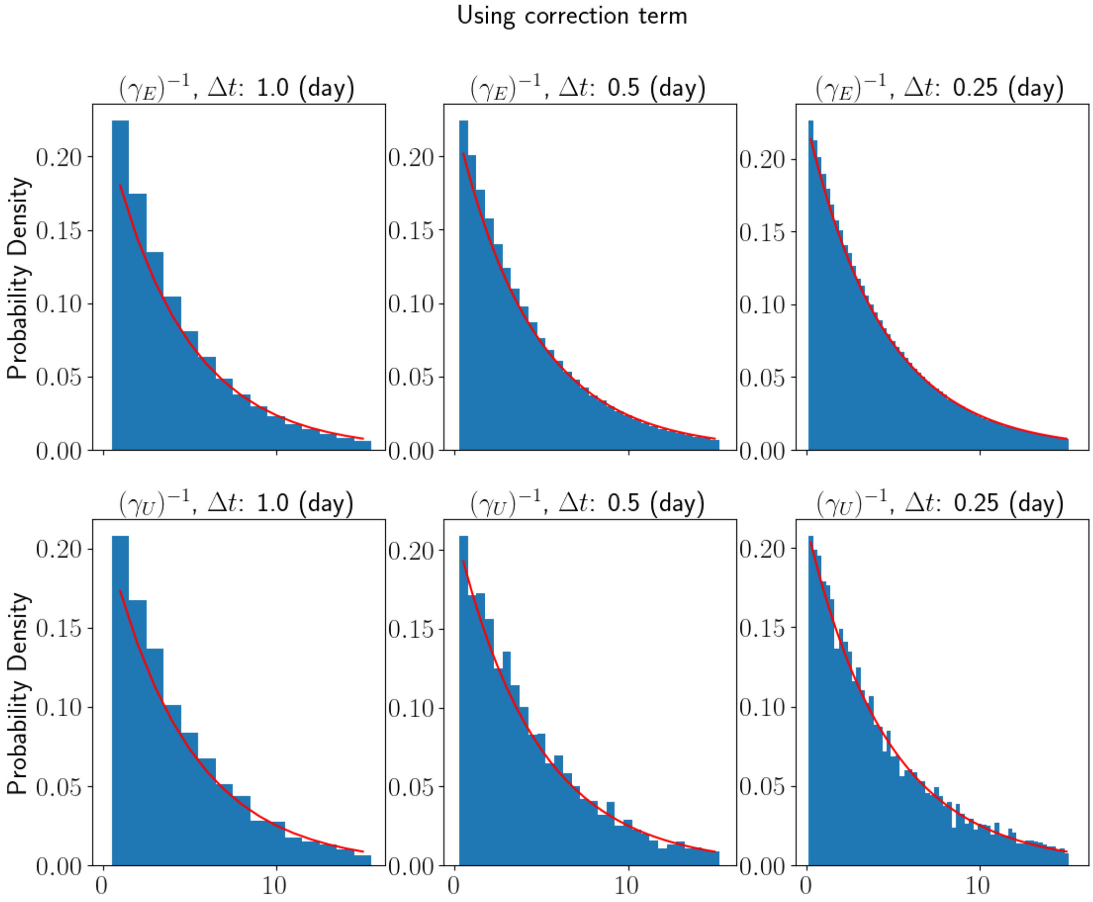

Figure 26: Frequency density of lengths of stay of agents in states E and U with the use of the correction term. The titles of each plot depict the associated parameter of the state in consideration and the time step (in days). The first row corresponds to lengths of time agents spend in the E state, which should correspond to an exponential distribution with mean γ_E, and the second row corresponds to lengths of time agents spend in the U state, which should correspond to an exponential distribution with mean γ_U. The first column uses a time unit of 1 day, the second column uses a time unit half a day and the last column uses a time unit of a quarter of a day.

Table 5: Observed values, with percentile-ranges, of the probability parameters using a different number of Monte Carlo iterations to compare the observed value against the true value given the state and fitted parameter, where m_H is the proportion of individuals who die in hospital (calculated by $\mu_H(\gamma_H + \mu_H)^{-1}$).

	Monte Carlo iterations				
Parameter	1	5	10	20	True value
p	0.9413	0.9409<br>(0.9404, 0.9414)	0.9405<br>(0.9396, 0.9411)	0.9407<br>(0.9390, 0.9421)	0.9401
m_U	0.0013	0.0014<br>(0.0013, 0.0015)	0.0013<br>(0.0012, 0.0014)	0.0014<br>(0.0012, 0.0015)	0.0013
m_H	0.3847	0.3850<br>(0.3817, 0.3888)	0.3878<br>(0.3799, 0.3964)	0.3844<br>(0.3782, 0.3908)	0.3843

References

[1] Campillo-Funollet E, Van Yperen J, Allman P, Bell M, Beresford W, Clay J, et al. Predicting and forecasting the impact of local outbreaks of COVID-19: Use of SEIR-D quantitative epidemiological modelling for healthcare demand and capacity. Int J Epidemiol. 2021;50(4):1103–1113. doi:10.1093/ije/dyab106.

[2] UKHSA. Coronavirus (COVID-19): Using data to track the virus. 2020 Apr 23 [cited 19 Jan 2023]. In: UK Health Security Agency Blogs [Internet]. Available from: https://ukhsa.blog.gov.uk/2020/04/23/coronavirus-covid-19-using-data-to-track-the-virus/.

[3] Campillo-Funollet E, Wragg H, Van Yperen J, Duong DL, Madzvamuse A. Reformulating the susceptible–infectious–removed model in terms of the number of detected cases: well-posedness of the observational model. Philos Trans Royal Soc. 2022;380(2233):20210306. doi:10.1098/rsta.2021.0306.

[4] Albani V, Loria J, Massad E, Zubelli J. COVID-19 underreporting and its impact on vaccination strategies. BMC Infect Dis. 2021;21(1):1–13. doi:10.1186/s12879-021-06780-7.

[5] Lau H, Khosrawipour T, Kocbach P, Ichii H, Bania J, Khosrawipour V. Evaluating the massive underreporting and undertesting of COVID-19 cases in multiple global epicenters. Pulmonology. 2021;27(2):110–115. doi:10.1016/j.pulmoe.2020.05.015.

[6] Gibbons CL, Mangen MJJ, Plass D, Havelaar AH, Brooke RJ, Kramarz P, et al. Measuring underreporting and under-ascertainment in infectious disease datasets: a comparison of methods. BMC Public Health. 2014;14(1):1–17. doi:10.1186/1471-2458-14-147.

[7] Chandra RV, Varanasi BS. Python requests essentials. Packt Publishing Ltd; 2015.

[8] McKinney W, et al. Data structures for statistical computing in python. In: van der Walt S, Millman J, editors. Proceedings of the 9th Python in Science Conference. vol. 445; 2010. p. 51–56.
